# *Aucklandia lappa* Decne.: a review of its botany, cultivation, ethnopharmacology, phytochemistry, pharmacology, and practical applications

**DOI:** 10.3389/fphar.2025.1659831

**Published:** 2025-10-13

**Authors:** Jing Chen, Zilong Zhao, Lihua Lin, Guangyao Wang, Haixia Yang, Xinghua Wang

**Affiliations:** ^1^ School of Environmental and Food Engineering, Liuzhou Polytechnic University, Liuzhou, China; ^2^ School of Chemical Engineering, Northwest University, Xi’an, China; ^3^ College of Traditional Chinese Medicine, Nanjing University of Chinese Medicine, Nanjing, China; ^4^ Brain Hospital Affiliated to Nanjing Medical University, Nanjing, China; ^5^ Faculty of Medicine and Health Sciences, Universiti Tunku Abdul Rahman, Selangor, Malaysia

**Keywords:** *Aucklandia lappa* Decne., *Aucklandiae* Radix, Chinese medicine, applications, Muxiang (Radix Aucklandiae)

## Abstract

*Aucklandia lappa* Decne. (ALD), a synonym of *Saussurea costus* (Falc.) Lipsch., is a traditional Chinese medicinal herb extensively cultivated in China. *Aucklandiae* Radix (AR, known as “Muxiang” in China), derived from the dried root of ALD, holds a significant position in the clinical application of traditional Chinese medicine, encompassing the enhancement of gastrointestinal motility, antibacterial properties, and antitumor activities. Notably, AR possesses a complex and diverse chemical composition, with costunolide and dehydrocostus lactone being its core active metabolites. This review provides an in-depth exploration of the biological characteristics, cultivation techniques, ethnopharmacology, phytochemistry, pharmacological activities, and processing techniques associated with ALD. To collect relevant research materials, the study systematically retrieved information from authoritative databases such as CNKI, PubMed, Elsevier, Web of Science, and SpringerLink, employing keywords including “cultivation,” “phytochemistry,” “pharmacology,” and the plant names “*Aucklandia lappa* Decne.,” “*Saussurea costus* (Falc.) Lipsch.,” or “*Aucklandiae* Radix.” Despite demonstrating remarkable pharmacological activities and potential for clinical applications, research on ALD still faces several challenges. For instance, its specific mechanisms of action in treating certain diseases remain incompletely understood, and multiple studies have indicated that ALD extracts may cause adverse reactions. Further in-depth research and systematic evaluation can facilitate the optimization of ALD practices to promote further research into its myriad applications.

## 1 Introduction

*Aucklandia lappa* Decne. (ALD), synonym of *Saussurea costus* (Falc.) Lipsch., is a perennial herb belonging to the Asteraceae family that possesses a rich medicinal history extending over two millennia (Huang et al., 2021; Song et al., 2021). Currently, ALD is predominantly cultivated in the Yunnan, Guizhou, Guangxi and Sichuan provinces of China (Xue et al., 2020). *Aucklandiae* Radix (AR), commonly known as Muxiang or Yunmuxiang (Chinese trade name), is derived from the dried root of ALD. It has been utilized extensively as a medicinal material in traditional Chinese medicine (TCM) and is officially recognized in the Chinese Pharmacopoeia (Huang et al., 2021; Shum et al., 2007). Since 2014, when related industrial bases were established in Yunnan and Guizhou Province and Chongqing of China, large-scale planting was carried out based on the growth characteristics of ALD, and its economic benefits have achieved a leapfrog growth. In 2021, the national sales volume of ALD was approximately 6,000 tons, and by 2024, the national sales volume of ALD exceeded 8,300 tons.

The pharmacological effects of AR are diverse and significant (Li S.-Y. et al., 2024). Modern pharmacological researches have demonstrated that AR exhibits several properties, including the promotion of gastrointestinal motility, dilation of bronchial smooth muscles, antibacterial activity, reduction of blood glucose levels, and antitumor effects (Song et al., 2022b; Zhang et al., 2021; Zheng et al., 2022; Zhuang et al., 2021). The chemical composition of AR is complex and diverse, primarily encompassing terpenoids, glycosides, and other metabolites (anthraquinones, flavonoids, amino acids, etc.). Costunolide and dehydrocostus lactone are principal active metabolites in AR (Huang Z. et al., 2022). The total content of costunolide (PubChem CID 5281437, C_15_H_20_O_2_) and dehydrocostus lactone (PubChem CID 73174, C_15_H_18_O_2_) in AR must not be less than 1.8%, as stipulated by the Chinese Pharmacopoeia (2020 Edition). Concurrently, the European Union’s Traditional Herbal Medicinal Products Directive has officially recognized AR as a certified herbal preparation for the treating functional dyspepsia.

**FIGURE 1:**
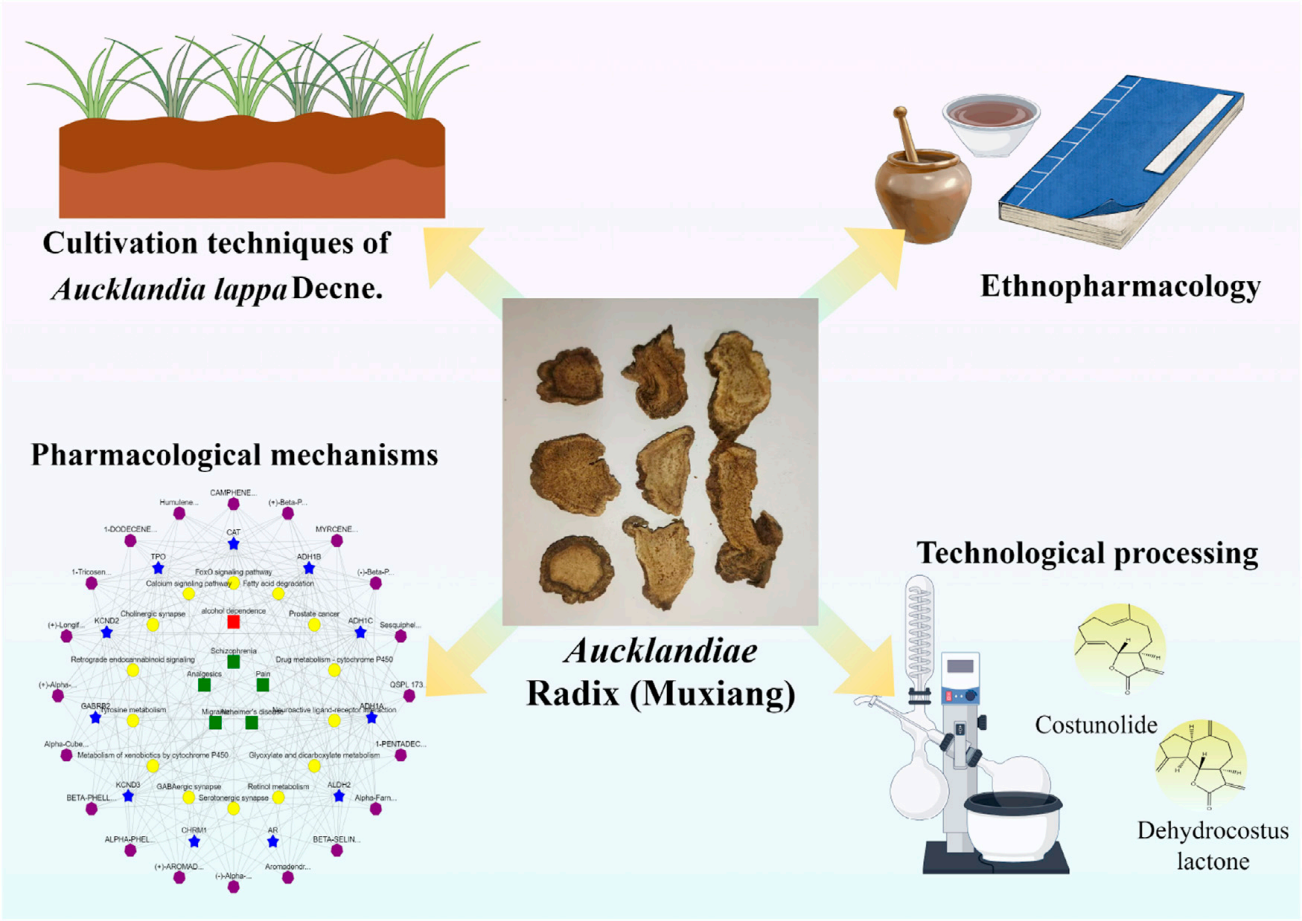
A. Radix (by figdraw.com).

This review provides a comprehensive examination of the biological characteristics, cultivation techniques, chemical composition, traditional Chinese medicinal applications, pharmacological activities, and processing methods associated with ALD (Figure 1). Large-scale cultivation and standardized production, circular economy model innovation, and the development of bio-synthesized active substances along with alternative pathways can facilitate the optimization of ALD practices to promote further research into its diverse applications.

## 2 Botanical characteristics

ALD is a perennial, tall herb characterized by a thick main root with a distinctive aroma. The basal leaves are triangular-ovate, featuring pinnately lobed upper long petioles and shallowly lobed margins. The stem leaves are also triangular-ovate or ovate, with the base extending downward and being sessile or having winged petioles. ALD produces capitula with multiple outer bracts, and its flowers are bisexual, dark purple, tubular, with inferior ovaries. The flowering period occurs from July to August, followed by a fruiting period from August to September (Chen, 2025). ALD thrives in cool to cold climates and exhibits significant cold resistance, making it particularly suitable for high-altitude regions with relatively low temperatures and high humidity. This species is a deep-rooted plant, with roots extending 30–50 cm or even deeper into the soil.

## 3 Cultivation and management

The cultivation of ALD plays a decisive role in determining the quality of its medicinal material. This influence is primarily reflected in four key aspects: geo-authenticity, growing environment, cultivation management, and harvesting and processing practices. Firstly, the specific altitude, climate, and soil conditions in geo-authentic regions promote the accumulation of higher levels of active metabolites (such as costunolide and dehydrocostus lactone) in the plant. In contrast, non-authentic producing areas typically yield inferior medicinal efficacy. Secondly, scientific cultivation management is central to quality assurance. The use of high-quality seeds, emphasis on well-rotted organic fertilizers, crop rotation, and integrated green pest management ensure healthy plant growth without pesticide residues. Conversely, excessive use of chemical fertilizers and pesticides compromises quality and introduces safety risks. Finally, timely harvesting and standardized post-harvest processing are crucial for preserving the therapeutic potency and aromatic properties. Harvesting too early or too late, or using high-temperature drying methods, can lead to the loss of volatile oils and significantly diminish efficacy. Therefore, standardized management throughout the entire process, is essential for producing ALD that is safe, effective, and of high quality.

### 3.1 Cultivation techniques

When selecting a cultivation site for ALD, it is imperative to ensure that the slope faces east or north and the slope is maintained at 30°–35°. A shaded slope is preferable. Additionally, the soil should be loose, fertile and possess a deep profile, with loam or sandy loam enriched with humus being optimal, as this composition facilitates effective drainage. Low-lying areas, which are susceptible to flooding and consequently to root rot, should be avoided. For newly developed land, it is essential to thoroughly clear all the weeds and existing vegetation from the wasteland, incorporating these materials into the soil through deep plowing. Deep plowing should be carried out again in the following spring. In addition, cultivators may opt to grow wood-scented plants on land that has previously supported crops such as potatoes and corn (Chen, 2025). Given that ALD can accumulate harmful metals from its native soil, consumption of low-quality ALD may lead to the accumulation of toxic metal elements in the human body (Meng et al., 2021). Inductively coupled plasma mass spectrometry (ICPMS) provides a precise and reliable method for monitoring and controlling contamination levels in the extract, such as Cu, Pb, As, Cd, and Hg in ALD (Chen et al., 2023). The soil used for planting should effectively control the pollution of related metals. The arbuscular mycorrhizal fungi strains (*Gigaspora decipiens*, *Scutellospora calospora*, *Racocetra coralloidea*, *Septoglomus deserticola*, *Entrophospora colombiana*, *Paraglomus brasilianum*), which had a good symbiotic relationship with ALD, are the potential strains to inoculate ALD seedlings under artificial cultivation conditions (Yang et al., 2020). Symbiotic bacteria can significantly increase terpenoids accumulation (costunolide, dehydrocostus lactone, etc.), soluble protein, soluble sugar, antioxidant enzyme activity in the leaves.

### 3.2 Reproductive methods

Seed propagation serves as the primary method for the cultivation of ALD. In Yunnan Province, the cultivation of ALD predominantly involves seeds sowing, which can be conducted during the winter, autumn, and spring. Typically, spring sowing occurs from mid-March to early April, autumn sowing from late August to mid-September, and winter sowing in early November. Post-harvest, the seeds must be sun-dried and subsequently cleaned of impurities to prepare them for subsequent processes. Prior to sowing, it is essential to prepare the seeds by soaking them in lukewarm water with continuous stirring. As the water cools, impurities and non-viable seeds are removed, leaving only the viable seeds that settle at the bottom. These viable seeds are then soaked for 24 h before being partially dried in preparation for sowing.

If the seeds quantity is insufficient for planting requirements, asexual reproduction can be considered. It is crucial to avoid using fine roots with medicinal value as breeding material. During planting, the layout should be carried out according to the prescribed spacing. And when covering the soil, it is necessary to ensure that the root systems are completely and tightly buried. ALD propagation via cuttings requires a healthy selection of semi-hardened stems with 2–3 nodes. Stems are trimmed to retain 2–3 leaves for photosynthesis, followed by treatment with rooting hormones or basal soaking in diluted rooting agent for 30 min prior to air-drying. A sterile, well-draining substrate (e.g., a mixture of leaf mould and perlite) is prepared and sterilized to minimize pathogen risk. Processed cuttings are inserted into the substrate at a depth of 1/3–1/2 the stem length. Post-insertion, the medium is moderately watered to maintain moisture without waterlogging. Environmental conditions are maintained at 20 °C–25 °C with indirect light and adequate airflow to avoid direct sunlight exposure. Rooting initiates within 3–4 weeks, accompanied by new shoot development.

Large-scale planting has been carried out based on the growth characteristics of ALD in Yunnan and Guizhou Province and Chongqing of China. This large-scale planting is still mainly based on traditional agricultural cultivation methods. In the future, the yield, quality and production efficiency of ALD can be improved through multi-disciplinary means such as micropropagation and tissue culture technology, molecular biology, intelligent equipment and environmental control. Micropropagation and tissue culture technology represent a fundamental advancement in contemporary crop cultivation (Alanagh et al., 2014; Lu et al., 2019). By employing asexual reproduction and cellular engineering techniques, these methods effectively address the challenges of low efficiency and genetic instability inherent in traditional seed propagation (Pupilli and Barcaccia, 2012). The root tips of ALD serve as highly differentiated potential explants suitable for plant tissue culture. By precisely regulating the growth system through genetic modification and improving stress resistance and the targeted accumulation of metabolites, the production efficiency and product value of ALD can be significantly enhanced. The precise regulation of nutrient solutions during the cultivation process can now be easily implemented (Lu et al., 2022; Wang et al., 2023). The demand for elements such as nitrogen, phosphorus, potassium, and calcium varies significantly at different stages of plant growth. Through dynamic monitoring and intelligent intervention, the accumulation of secondary metabolites, stress resistance, and growth consistency of medicinal plants like ALD may be significantly improved. Furthermore, based on historical data, a growth prediction model for ALD can be constructed, integrating environmental variables (temperature, precipitation, soil EC value) to output the best irrigation and fertilization decisions. If synthetic biology and intelligent equipment can be effectively combined, the ALD industry is expected to become a benchmark model in the global medicinal plant field.

### 3.3 Control of pests and diseases

Leaf spot disease and root rot represent significant threats to the growth cycle of ALD, particularly during the rainy season when the prevalence of these diseases increases markedly, with July and August identified as peak periods. To mitigate these challenges, growers should prioritize land with superior drainage and a lower groundwater table for cultivating ALD. During field management, it is crucial to handle the plants carefully to prevent root injury and to rigorously implement quarantine measures to ensure that seeds are free from pathogens. Once infected plants are identified, they should be immediately removed, and the soil should be disinfected with quicklime to curb the further spread of root rot (Tan et al., 2023). In cases where ALD seedlings exhibit symptoms of root rot, growers need to take prompt action by precisely spraying an appropriate amount of thiophanate-methyl or carbendazim on the roots of the affected plants (Gaitnieks et al., 2016). Additionally, during the rainy season, the application of Bordeaux mixture on clear days is advised to effectively prevent leaf spot disease. Upon noticing symptoms of disease on ALD seedlings, an appropriate amount of bactericides (tebuconazole or chlorothalonil) should be evenly sprayed on the leaves, with attention to rotating different pesticides to enhance control effectiveness.

The primary pests impacting ALD include grasshoppers, aphids, cutworms, and grubs. During their nymphal stage, grasshoppers can be effectively managed by spraying with a solution of 90% crystalline trichlorfon diluted to 800 times (Zhang et al., 2019). Aphids can be controlled by spraying with a solution of 40% dimethoate emulsifiable concentrate diluted 800 to 1,500 times (Chandrasena et al., 2011). Cutworms and grubs, which damage seedlings, roots, and leaves, can be effectively trapped and eradicated using bait made by combining crystalline trichlorfon with wheat bran (Kumar and Pandey, 2022; Smirle et al., 2013).

## 4 Ethnopharmacology

The earliest documentation of AR appears in the ancient book “Supplements to the *Shennong Bencao* by Medical Masters” (《名医别录》). Subsequently, classical works such as “The Newly Revised Materia Medica” (《新修本草》), “Illustrated Classic of Materia Medica” (《本草图经》), “Essential Documents of the Tang Dynasty” (《唐会要》), “Muslim Medicine” (《回回药方》), and “Compendium of Materia Medica” (《本草纲目》) have systematically recorded its applications, underscoring its integration into both Chinese pharmacology and cross-cultural medical traditions. In the Qing Dynasty, the “Essential Compendium of Materia Medica” (《本草备要》) systematically standardized the processing techniques of ALD, establishing protocols that balanced traditional practices with empirical refinement. At present, it is officially recognized in the Chinese Pharmacopoeia (《中国药典》2020 Edition).

### 4.1 Identification of AR

AR originates from the dried root of ALD, which is collected during the autumn and winter seasons. Once dried, the coarse outer skin is removed. The final product is typically cylindrical or semi-cylindrical, measuring between 5 and 10 cm in length and 0.5–5 cm in diameter. Its surface boasts a yellowish-brown to grayish-brown coloration, adorned with prominent wrinkles, longitudinal grooves, and lateral root marks. The texture is notably firm, making it resistant to breaking. Upon sectioning, the interior reveals a grayish-brown to dark-brown coloration, with a grayish-yellow or light brownish-yellow periphery. A distinct brown cambium ring is evident, accompanied by a radial texture. It possesses a unique aroma and a slightly bitter taste (China Pharmacopoeia Committee, 2020a).

### 4.2 Traditional uses of AR

Four traditional AR-based prescriptions have been recorded in the Chinese Pharmacopoeia (2020 edition): Muxiang Fenqi Wan (China Pharmacopoeia Committee, 2020e), Muxiang Shunqi Wan (Bai, 2023; China Pharmacopoeia Committee, 2020f), Muxiang Binglang Wan (China Pharmacopoeia Committee, 2020d; Gao et al., 2024), and Liuwei Muxiang San (China Pharmacopoeia Committee, 2020c). These traditional medicines primarily treat gastrointestinal stagnation, abdominal distension pain, and bowel obstruction.

Additionally, other traditional prescriptions that were not included in the Chinese Pharmacopoeia have also been studied (Table 1). Simo Decoction has been shown to enhance gastrointestinal motility by influencing the contractions of antral circular smooth muscle strips (Dai et al., 2012). JianPi’I, which originates from the classical Chinese medicine formula Xiangshaliujunzi decoction, is known to alleviate symptoms associated with postprandial distress syndrome (Wang et al., 2016). The Xian-He-Cao-Chang-Yan formula has been demonstrated to ameliorate DSS-induced colitis in mice (Li J. et al., 2021). Weichang’an Pill has been widely used for decades in the treatment of irritable bowel syndrome and functional dyspepsia (Zhang et al., 2013). Kangen-karyu (GuanYuan-Ke-Li) is considered a promising therapeutic agent for Alzheimer’s disease (Paudel et al., 2020). Xianglian Pill, composed of *Rhizoma coptidis* and AR, has long been employed in the management of gastrointestinal disorders (Lu et al., 2020). Clinically, Wuwei Shexiang Pill is used in China to reduce joint pain and swelling, as well as to dispel wind and alleviate pain (Bai et al., 2023). Additionally, treatment with Muxiang gel plaster has been found to effectively prevent and mitigate mammary hyperplasia (Wu Y. et al., 2024).

**TABLE 1:** List of TCM prescriptions related to ALD.

Name	Formula (except for AR)	Indications	Ref.
Muxiang Fenqi Wan	*Wurfbainia compacta*, *Syzygium aromaticum* L. , *Santalum album*, *Cyperus rotundus* L., *Pogostemon cablin*, *Citrus reticulata* Blanco, *Magnolia officinalis*, *Citrus aurantium* L., *Curcuma longa* L., *Crataegus monogyna* Jacq., *Atractylodes macrocephala* Koidz., *Nardostachys jatamansi*, *Areca catechu* L., *Glycyrrhiza glabra* L.	Bloating, abdominal pain	China Pharmacopoeia Committee (2020e)
Muxiang Shunqi Wan	*Wurfbainia compacta*, *Cyperus rotundus* L., *Areca catechu* L., *Glycyrrhiza glabra* L., *Magnolia officinalis*, *Citrus aurantium* L., *Atractylodes Lancea*, *Citrus reticulata* Blanco, *Zingiber officinale* Roscoe	Bloating, abdominal pain	(Bai, 2023; China Pharmacopoeia Committee, 2020f)
Muxiang Binglang Wan	*Areca catechu* L., *Citrus aurantium* L., *Citrus reticulata* Blanco, *Cyperus rotundus* L., *Sparganium emersum* Rehmann, *Curcuma longa* L., *Coptis chinensis* Franch., *Cortex Phellodendri* Chinensis, *Rheum officinale* Baill., *Semen Pharbitidis*, *Natrii Sulfas*	Bloating, abdominal pain, uncomfortable bowel movements	(China Pharmacopoeia Committee, 2020d; Gao et al., 2024)
Liuwei Muxiang San (Wan)	*Gardenia jasminoides*, *Punica Granatum*, *Rhododendron molle*, *Wurfbainia compacta* Rotundus, *Piper longum* L.	Bloating, abdominal pain, uncomfortable bowel movements	(China Pharmacopoeia Committee, 2020c; Renqing-Dongzhu et al., 2023)
Simo decoction	*Citrus aurantium* L., *Areca catechu* L., *Lindera aggregata* (Sims) Kosterm.	Gastrointestinal dysmotility	(Dai et al., 2012; Yi et al., 2011)
JianPi'I	*Codonopsis pilosula* (Franch.) Nannf., *Atractylodes macrocephala* Koidz., *Poria cocos*, *Wurfbainia compacta*, *Glycyrrhiza glabra* L., *Citrus reticulata* Blanco, *Pinellia ternata* (Thunb.) Makino	Postprandial distress syndrome	Wang et al. (2016)
Xian-He-Cao-Chang-Yan formula	*Agrimonia eupatoria* L., *Coptis chinensis* Franch., *Cyperus esculentus* L., *Acorus verus* (L.) Raf., *Platycodon grandiflorus* Jacq.	Colitis	Li et al. (2021a)
Weichang’an Pill	*Lignum Aquilariae Resinatum*, *Lignum Aantali Albi*, *Citrus aurantium* L., *Cortex Magnoliae officinalis*, *Rheum officinale* Baill., *Conioselinum anthriscoides*, *Croton tiglium* L., *Ziziphus jujuba* Mill., *Abelmoschus moschatus* Medik.	Irritable bowel syndrome and functional dyspepsia	Zhang et al. (2013)
Kangen-karyu (GuanYuan-Ke-Li)	*Salvia miltiorrhiza* Bunge, *Conioselinum anthriscoides*, *Paeonia lactiflora* Pall., *Carthamus tinctorius* L., *Cyperus rotundus* L.	Alzheimer’s disease	Paudel et al. (2020)
Xianglian pill	*Coptis chinensis* Franch.	Gastrointestinal disease	Lu et al. (2020)
Wuwei Shexiang pills	*Terminalia chebula* Retz., *Abelmoschus moschatus* Medik., *Aconitum carmichaelii* Debx., *Acorus calamus* L.	Joint pain	Bai et al. (2023)
Muxiang gel plaster (Muxiang Bing)	Edible gelatin, *Rehmannia glutinosa* (Gaertn.) Libosch., catalpol, rehmannioside D, tartaric acid, carbomer, sodium polyacrylate, dihydroxyaluminium aminoacetate, glycerol	Mammary hyperplasia	Wu et al. (2024b)
Anshen-Buxin-Liuwei pill	*Bos taurus domesticus Gmelin*, *Choerospondias axillaris*, *Myristica fragrans* Houtt., *Eugenia caryophμllata* Thunb., *Liquidambar formosana*	Cardiomyocyte hypoxia/reoxygenation injury	Huang et al. (2022a)

With the development of TCM, the active metabolites in medicinal substances can be concentrated, extracted and combined to enhance their therapeutic efficacy. A metabolite formulation, consisting of three herbal extracts (CO_2_ supercritical fluid extract of ginger, ethanol reflux extract of AR, and pogostemonis herba essential oil) has been developed as a promising anti-motion sickness treatment (Zhang et al., 2015).

### 4.3 Traditional processing

The traditional processing techniques for ALD involves several methods aimed at enhancing its efficacy and adaptability for various Chinese medicinal formulations (Li X. et al., 2021; Song et al., 2022a). These methods include the use of raw AR, stir-fried AR, baked (or grilled) AR, and wine-processed AR. Raw AR denotes the cleaned and dried form of the original medicinal material, which undergoes impurity removal, washing, slicing, and drying. Stir-fried AR is prepared by lightly frying the slices with bran before they are used in formulations. Baked AR, also known as grilled AR, involves stir-frying the pieces with bran until they turn yellow, after which they are cooled and incorporated into medicinal applications (Wu M.-l. et al., 2024). Wine-processed AR is made by moistening the botanical drug with rice wine, followed by slicing and drying for medicinal use.

These traditional processing techniques for the botanical drug AR significantly influence the quality and therapeutic direction of the final product by carefully controlling the heating intensity and the use of auxiliary materials: raw AR retains abundant volatile oils, offering strong medicinal properties but also greater side effects due to alkaloids and other metabolites; roasting gently heats the botanical drug at low temperatures, promoting the release or transformation of some volatile oils, thereby reducing side effects and mildly enhancing its gastrointestinal therapeutic functions; plain stir-frying and bran-frying reduce toxicity and adverse effects while strengthening its therapeutic efficacy; wine-processing using rice wine facilitates the extraction of active metabolites, increasing pharmacological activity. Essentially, these methods work by regulating the content and transformation of volatile oils and other metabolites to achieve reduced toxicity, preserved efficacy, enhanced potency, or altered therapeutic targeting (Feng et al., 2023; Li R.-l. et al., 2021).

### 4.4 Adverse reaction (toxicity)

Although AR has good medicinal value, excessive use may lead to adverse effects. Skin allergic reaction is a common side effect in the use of AR, due to various irritant alkaloids (saussureanine, costunolide, etc.), for people who are sensitive or have existing skin inflammation, contact with these substances is easy to cause allergic reactions. Acute generalized exanthematous pustulosis also could induced by AR (Chu et al., 2019). Additionally, high doses of TCM usually have liver toxicity, and AR is no exception. Oxidative stress should be the primary mechanism for the high-dose AR-induced hepatotoxicity, and Nrf2, HO-1 and NQO1 were the main targets (Song et al., 2024). On the contrary, the appropriate dosage of ethanol extract of AR had a protective effect on liver injury induced by lipopolysaccharide in rats (Song et al., 2023). Reasonable control of the dosage of AR is important for patients to avoid side effects, which can effectively ensure the safety and effectiveness of Chinese herbal medicine.

### 4.5 Commonly confused botanical drugs

In the field of traditional botanical drugs, species within the AR, *Aristolochiae* Radix (ARR), *Vladimiriae* Radix (VR), and *Inulae* Radix (IR), present persistent identification challenges due to morphological convergence and historical misclassification (Zhuang et al., 2021). These taxonomically related species exhibit overlapping botanical characteristics, particularly in root morphology and histological features, resulting in frequent substitution errors in herbal commerce. Current pharmacognostic authentication relies on UHPLC-QTOF-MS as the gold-standard methodology for precise differentiation (Guccione et al., 2017; Shum et al., 2007; Wang et al., 2024). This analytical approach enables simultaneous detection of multiple marker metabolites, achieving clear discrimination between structurally analogous aristolochic acid derivatives and sesquiterpene lactone.

VR (“Chuanmuxiang” in China), primarily derived from the dried roots of *Vladimiria souliei* (Franch.) Ling or *V. souliei* (Franch.) Lingvar. *cinerea* Ling, predominantly cultivated in western Sichuan, China, exhibits medicinal properties akin to those of Muxiang, albeit with distinct emphases (China Pharmacopoeia Committee, 2020b). Chemical profiling differentiates AR from VR based on significant differences in their quantitative composition (dihydrodehydrocostus lactone, mokkolactone, α-costol, etc.) (Chen et al., 2020; Liang et al., 2024; Yan et al., 2020). The total lactone extract of VR has the similar protective effects on cholestatic liver injury as AR, even better in terms of anti-inflammatory properties (Chen Z. et al., 2022).

IR (“Tumuxiang” in China) refers to the dried roots of *Inula helenium* L. or *Inula racemosa* Hook.f. (China Pharmacopoeia Committee, 2020g). Historically utilized as a substitute for AR, IR exhibits significant chemical distinctions from AR, with its primary differential metabolites being alantolactone and isoalantolactone (Cai et al., 2021; Rasul et al., 2013). These metabolites have been used to emesis and diarrhoea, and to eliminate parasites (Xu et al., 2015). Alantolactone and isoalantolactone have been confirmed to possess potential hepatotoxicity, nephrotoxicity, and allergenic effects. Long-term or excessive intake may lead to symptoms such as loss of appetite, fatigue, nausea, vomiting, and pain in the liver area. In severe cases, it can cause irreversible liver damage. Due to their toxicity, IR has been rarely used in modern clinical practice of TCM.

ARR (“Qingmuxiang” in China) is the dried roots of *Aristolochia debilis* Sieb. et Zucc. or *Aristolochia contorta* Bge. ARR originally appeared as a substitute for AR, but contemporary researches have revealed the presence of aristolochic acid in *Aristolochiae* species, which can cause nephrotoxic adverse reactions (Cheng et al., 2006; Wang X. et al., 2020; Zhang et al., 2016). Consequently, ARR has been banned from use in TCM formulations (Luo et al., 2024).

In summary, careful identification of AR’s species authenticity and quality is essential during procurement and use to prevent confusion with commonly confused botanical drugs. At the same time, it is recommended to use AR and its related botanical drugs under the guidance of a professional physician or pharmacist.

### 4.6 Botanical drugs with similar therapeutic properties

The commercially available herbal medicines exhibiting comparable qi-regulating, analgesic, spleen-strengthening, and digestion-promoting activities to AR primarily include *Pericarpium Citri Reticulatae*, *Aurantii Fructus*, *Cyperi Rhizoma*, and *Amomi Fructus*. However, their therapeutic emphases and clinical applications exhibit notably divergent profiles. Additionally, AR demonstrates multidimensional industrial advantages over other botanical drugs in its category.

Specifically, the flavonoid metabolites (hesperidin, nobiletin, tangeretin, etc.) in *Pericarpium Citri Reticulatae* are susceptible to rapid degradation under UV light, resulting in poor stability (Ho and Kuo, 2014; Li Y. et al., 2024). *Aurantii Fructus* contains alkaloids such as p-tyramine, N-methyltyramine, and tyramine that may cause cardiovascular adverse reactions including hypertension and arrhythmia, thus requiring careful evaluation of the patient’s baseline condition in clinical use (Gao et al., 2020). Cyperus volatile oils, rich in α-cyperone, cyperotundone, and limonene, face industrial scalability challenges due to low extraction yields (Bezerra et al., 2025). *Amomi Fructus* is constrained by its reliance on specific cultivation conditions and high production costs (Suo et al., 2018). In contrast, AR not only has a mild nature and comprehensive effects, but also stands out in terms of the stability of its metabolites, safety, and the maturity of industrialization. Therefore, it is more suitable for large-scale pharmaceutical production, food processing, and the development of health products.

## 5 Phytochemistry

Currently, more than 200 metabolites (Table 2) have been isolated and identified from ALD (Huang et al., 2021; Zheng et al., 2022). These metabolites can be categorized by structural type into sesquiterpene lactones (eudesmanolides, guaianolides, germacranolides, etc.), monoterpenoids, triterpenoids, phenylpropanoids, steroids, flavonoids, amino acids, and other metabolites (Huang et al., 2021; Seo et al., 2015; Wang Y. et al., 2020; Zheng et al., 2022). These metabolites were primarily isolated and identified using techniques such as thin-layer chromatography (TLC), nuclear magnetic resonance (NMR), liquid chromatography (LC), gas chromatography (GC), mass spectrometry (MS), and electronic nose (e-nose) technology.

**TABLE 2:** Main metabolites isolated from AR (Zheng et al., 2022; Zhuang et al., 2021).

Number	Classification	Molecular formula	Metabolites
1	Sesquiterpene lactones - eudesmanolides	C_15_H_20_O_2_	α-Cyclocostunolide
2		C_15_H_20_O_3_	Santamarine
3		C_15_H_22_O_3_	11α,13-Dihydrosantamarine
4		C_15_H_20_O_2_	β-Cyclocostunolide
5		C_15_H_20_O_3_	Reynosin
6		C_15_H_22_O_3_	11α,13-Dihydroreynosin
7		C_15_H_20_O_3_	Magnolialide
8		C_15_H_22_O_3_	11α,13-Dihydromagnolialide
9		C_15_H_22_O_3_	Arbusculin A
10		C_15_H_22_O_4_	1β-Hydroxyarbusculin A
11		C_15_H_20_O_2_	Arbusculin B
12		C_15_H_24_O_3_	Colartin
13		C_15_H_20_O_4_	1β-Hydroxycolartin
14		C_15_H_20_O_2_	Alantolactone
15		C_15_H_20_O_2_	Isoalantolactone
16		C_15_H_20_O_3_	Saussureal
17		C_15_H_22_O_2_	Dihydro-α-cyclocostunolide
18		C_20_H_28_NO_5_	Saussureamine D
19		C_20_H_28_NO_5_	Saussureamine E
20		C_16_H_22_O_6_S	13-Sulfo-dihydrosantamarine
21		C_16_H_22_O_6_S	13-Sulfo-dihydroreynosin
22	Sesquiterpene lactones - guaianolides	C_15_H_18_O_2_	Dehydrocostus lactone
23		C_15_H_20_O_2_	Dihydrodehydrocostus lactone
24		C_15_H_20_O_3_	4β-Methoxy-dehydrocostuslactone
25		C_15_H_20_O_3_	4α-Methoxy-dehydrocostuslactone
26		C_15_H_20_O_3_	10α-Methoxy-dehydrocostuslactone
27		C_15_H_18_O_3_	11,13-Epoxy-dehydrocostuslactone
28		C_15_H_16_O_4_	11,13-Epoxy-3-keto-dehydrocostuslactone
29		C_15_H_20_O_2_	Mokko lactone
30		C_16_H_22_O_3_	13-Methoxy-dihydrodehydrocostuslactone
31		C_17_H_22_O_4_	Lappalone
32		C_15_H_18_O_3_	Zaluzanin C
33		C_17_H_20_O_4_	Zaluzanin D
34		C_21_H_30_O_8_	11β,13-Dihydroglucozalunin C
35		C_15_H_18_O_3_	Isozaluzanin C
36		C_15_H_20_O_3_	11β,13-dihydro-3-epizaluzanin C
37		C_15_H_18_O_4_	11,13-Epoxyisozaluzanin C
38		C_15_H_18_O_2_	Isodehydrocostuslactone
39		C_15_H_16_O_3_	Isodehydrocostuslactone-15-aldehyde
40		C_15_H_16_O_3_	Isodehydrocostuslactone-14β- aldehyde
41		C_20_H_26_NO_4_	Saussureamine B
42		C_19_H_24_NO_5_	Saussureamine C
43		C_15_H_20_O_5_S	Sulfocostunolide A
44		C_18_H_20_O_6_	Cynaropicrin
45		C_15_H_20_O_5_S	Sulfocostunolide B
46		C_21_H_28_O_8_	3-O-β-D-Glucopyranoside-1α,3α,5α,7αH-guaiane-10(14),11 (13)-trien-6α,12-olide
47	Sesquiterpene lactones - germacranolides	C_15_H_20_O_2_	Costunolide
48		C_15_H_22_O_2_	11α,13-Dihydrocostunolide
49		C_15_H_22_O_2_	11β,13-Dihydrocostunolide
50		C_16_H_24_O_3_	13-Methoxydihydrocostunolide
51		C_20_H_28_NO_4_	Saussureamine A
52		C_21_H_30_O_8_	Picriside B
53		C_15_H_22_O_2_	Isodihydrocostunolide
54		C_15_H_20_O_3_	Soulangianolide A
55		C_15_H_20_O_3_	Parthenolide
56		C_20_H_26_O_6_	Eupatoriopicrin
57		C_15_H_22_O_2_	Saussurea lactone
58		C_20_H_30_O_8_	Saussurea lactone-10-O-β-D-glucoside
59		C_15_H_20_O_2_	Dehydrosaussurea lactone
60		C_30_H_38_O_6_	Lappadilactone
61	Other sesquiterpenoids	C_15_H_24_	α-Selinene
62		C_15_H_24_	β-Selinene
63		C_15_H_24_	γ-Selinene
64		C_15_H_24_O	α-Costol
65		C_15_H_24_O	β-Costol
66		C_15_H_24_O	γ-Costol
67		C_15_H_22_O	α-Costal
68		C_15_H_22_O	β-Costal
69		C_15_H_22_O	γ-Costal
70		C_15_H_22_O_2_	Costic acid
71		C_15_H_22_O_2_	Isocostic acid
72		C_15_H_26_O_2_	4β-Hydroxy-11 (13)-eudesmane-12-al
73		C_15_H_24_O_2_	Ilicol
74		C_15_H_22_O_3_	5α-Hydroxy-costic acid
75		C_15_H_26_O	γ-Eudesmol
76		C_15_H_26_O	α-Eudesmol
77		C_15_H_26_O	β-Eudesmol
78		C_15_H_24_	γ-Muurolene
79		C_15_H_24_	Eudesma-3,7 (11)-diene
80		C_17_H_26_O_4_	1β,6α-Dihydroxycostic acid ethyl ester
81		C_15_H_24_	β-Maaliene
82		C_15_H_24_	(+)-Germacrene
83		C_15_H_24_O	Germacra-1(10),4,11(13)-tiren-12-ol
84		C_15_H_22_O	Germacra-1(10),4,11(13)-tiren-12-al
85		C_15_H_22_O_2_	Germacra-1(10),4,11(13)-tiren-12-oic acid
86		C_15_H_24_	β-Elemene
87		C_15_H_24_O	Elema-1,3,1 (13)-tiren-12-ol
88		C_15_H_22_O	Elemenal
89		C_15_H_28_O	Elemol
90		C_15_H_24_	α-Humulene
91		C_15_H_24_	β-Humulene
92		C_15_H_24_	β-Caryophyllene
93		C_15_H_24_O	Epoxy-caryophyllene
94		C_15_H_24_	α-Cedrene
95		C_15_H_24_	β-Cedrene
96		C_15_H_24_O	Cedrenol
97		C_15_H_24_	α-Bergamotene
98		C_15_H_22_O	2,12-Bergamotadien-14-al
99		C_14_H_24_O	(E)-9-Isopropyl-6-methyl-5,9-decadien-2-one
100		C_15_H_20_	α-Calacorene
101		C_15_H_22_	α-Curcumene
102		C_15_H_24_	γ-Curcumene
103		C_15_H_24_	α-Zingiberene
104		C_15_H_24_	Valencene
105		C_15_H_24_	β-Sesquiphellandrene
106		C_15_H_24_O	Glaucyl alcohol
107		C_15_H_24_	β-Bergamotene
108		C_15_H_24_	Longifolene
109		C_15_H_24_	γ-Gurjunene
110		C_15_H_24_	α-Gurjunene
111		C_15_H_24_	Bisabolene
112		C_15_H_24_	cis-α-Bisabolene
113		C_15_H_30_O	Nerolidol
114		C_15_H_26_O	Viridiflorol
115		C_15_H_26_O	Globulol
116		C_15_H_26_O	Ledol
117		C_15_H_24_	α-Longipinene
118		C_15_H_24_	β-Guaiene
119		C_15_H_24_	α-Guaiene
120		C_15_H_22_O	Santalol
121		C_15_H_24_O	Aromadendrene epoxide
122		C_15_H_24_	trans-β-Farnesene
123		C_15_H_24_	α-Farnesene
124		C_15_H_24_	β-Himachalene
125		C_15_H_26_O	Hedycaryol
126		C_15_H_24_	Aromadendrene
127		C_15_H_22_O	Nootkatone
128		C_15_H_24_O	Thujopsanone
129		C_15_H_24_O	trans-α-Bergamotol
130		C_15_H_24_O	Santalcamphor
131		C_15_H_24_O	Longifolenaldehyde
132		C_15_H_24_O	Spathulenol
133		C_15_H_24_	α-Copaene
134		C_15_H_24_	α-Bulnesene
135		C_15_H_26_O	Valerianol
136		C_15_H_28_O_2_	Cryptomeridiol
137	Monoterpenoids	C_15_H_24_O	γ-Gurjunenepoxide-(2)
138		C_10_H_16_	Camphene
139		C_10_H_16_	Phellandrene
140		C_13_H_20_O	α-Ionone
141		C_13_H_20_O	β-Ionone
142		C_13_H_22_O	3,4-Dihydro-α-ionone
143		C_13_H_20_O_2_	(3R,6S)-α-Ionone-3-ol
144		C_13_H_17_O_2_	α-Ionone-4-one
145		C_13_H_20_O_2_	(3R,6R)-α-Ionone-3-ol
146		C_10_H_16_	Myrcene
147		C_10_H_18_O	Linalool
148		C_10_H_16_	α-Terpinene
149		C_10_H_16_	β-Terpinene
150		C_10_H_18_O	Isoborneol
151		C_10_H_18_O	Borneol
152		C_10_H_16_	γ-Terpinene
153		C_10_H_16_	α-Pinene
154		C_10_H_16_	β-Pinene
155		C_10_H_18_O	4-Terpineol
156		C_10_H_18_O	α-Terpineol
157		C_13_H_22_O	α-Ionol
158		C_10_H_16_	Limonene
159		C_10_H_16_	Terpinolene
160		C_13_H_22_O	Geranylacetone
161		C_10_H_20_O	Menthol
162		C_10_H_16_O	Carvotanacetone
163		C_12_H_22_O_2_	Linalyl acetate
164		C_10_H_18_O	Menthone
165		C_10_H_16_O	(+)-Camphor
166	Triterpenoids	C_30_H_50_O	α-Amyrin
167		C_34_H_58_O_2_	3β-Acetyl-9 (11)-baccharene
168		C_30_H_48_O_3_	Betulinic acid
169		C_30_H_50_O_2_	Betulinol
170		C_31_H_50_O_3_	Betulinic acid methyl ester
171		C_30_H_50_O	Taraxsterol
172		C_30_H_50_O	3-Filicanone
173	Phenylpropanoids	C_17_H_24_O_9_	Syringin
174		C_16_H_22_O_8_	4-Allyl-2,6-dimethoxyphenolglucoside
175		C_16_H_18_O_9_	Chlorogenic acid
176		C_10_H_12_O	Anethole
177		C_12_H_14_O_3_	Eugenol acetate
178		C_9_H_8_O	Cinnamaldehyde
179		C_10_H_10_O_2_	Safrole
180		C_10_H_12_O	Estragole/4-Allylanisole
181		C_12_H_8_O_4_	Bergapten
182		C_13_H_14_O_4_	6,8-Dimethoxy-3,7-dimethylisocoumarin
183		C_26_H_32_O_12_	1-Hydroxyrosinol-1-O-β-D-glucopyranoside
184		C_26_H_34_O_13_	(−)-olivil-4″-O-β-D-glucopyranoside
185		C_22_H_26_O_8_	Syringaresinol
186		C_20_H_22_O_8_	Prinsepiol
187		C_29_H_36_O_11_	(+) -1-Hydroxypinoresinol-4″-O-methyl ester-4′-β-D-glucopyranoside
188		C_28_H_34_O_10_	(+)-1-Hypinoresinol-4″-O-β-D-glucopyranoside
189	Steroids	C_30_H_46_O_6_	Lappalanasterol
190		C_31_H_50_O	3-Epi-lappasterol
191		C_21_H_32_O_2_	Pregnenolone
192		C_35_H_60_O_6_	Daucosterol
193		C_29_H_50_O	β-Sitosterol
194		C_29_H_48_O	Stigmasterol
195		C_28_H_48_O	Campesterol
196		C_29_H_50_O	γ-Sitosterol
197	Flavonoids	C_29_H_48_O_2_	Vlasoudiol
198		C_46_H_60_O_22_	3’-(3 R-Acetoxy-5,5-dimethylcyclopent-1-ene)-4′-Omethylscutellarein-7-O-(6‴’-Oacetyl-β-D-glucopyranosyl-(1 → 3)-[α-L-rhamnopyranosyl-(1 → 2)]-β-D-glucopyranoside
199		C_44_H_58_O_21_	Kaempferol-3-O-β-Dglucopyransoyl-(1 → 4)-α-Lrhamnopyranosyl-(1 → 6)-β-D-galactopyranoside 7-O-(6‴’O-acetyl-β-Dglucopyranosyl-(1 → 3)-[α-L-rhamnopyranosyl (1 → 2)]-β-D-glucopyranoside
200		C_47_H_54_O_20_	Kaempferol-3-O-β-Dglucopyranosyl (1 → 2)-β-D-(6a′-Ocaffeoyl)-glucopyranoside 7-O-(6‴ ’-O-acetyl-β-Dglucopyranosyl-(1 → 3)-[α-L-rhamno-pyranosyl-(1 → 2)]β-D-glucopyranoside
201		C_49_H_48_O_15_	Kaempferol-3-O-α-L-(2a′,3a′-E-di-p-coumaroyl)-rhamnoside 7-O-(6‴’-O-acetyl-β-Dglucopyranosyl-(1 → 3)-[α-Lrhamnopyranosyl-(1 → 2)]-β-D-glucopyranoside
202		C_10_H_10_O_4_	5,7-Dihydroxy-2-methylchromone
203		C_16_H_14_O_7_	1-Hydroxy-2,3,4,7-tetramethoxyxanthone
204		C_15_H_12_O_6_	1,7-Dihydroxy-3,4-dimethoxyxanthon
205	Amino acid	C_2_H_5_NO_2_	Aspartic acid
206		C_4_H_7_NO_4_	Glycine
207		C_4_H_8_N_2_O_3_	L-Asparagine
208		C_4_H_9_NO_2_	4-Aminobutyric acid
209		C_4_H_9_NO_3_	L-Threonine
210		C_3_H_7_NO_3_	Serine
211		C_5_H_9_NO_4_	Glutamic acid
212		C_5_H_10_N_2_O_3_	Glutamine
213		C_3_H_7_NO_2_	Alanine
214		C_6_H_13_N_3_O_3_	Citrulline
215		C_5_H_11_NO_2_	Valine
216		C_6_H_12_N_2_O_4_S_2_	Cystine
217		C_6_H_13_NO_2_	Isoleucine
218		C_6_H_13_NO_2_	Leucine
219		C_9_H_11_NO_3_	Tyrosine
220		C_9_H_11_NO_2_	Phenylalanine
221		C_5_H_12_N_2_O_2_	Ornithine
222		C_6_H_14_N_2_O_2_	Lysine
223		C_6_H_9_N_3_O_2_	Histidine
224		C_6_H_14_N_4_O_2_	Arginine
225		C_21_H_20_O_10_	Aloe-emodin-8-O-β-D-glucopyranoside
226		C_21_H_18_O_11_	Rhein-8-O-β-D-glucopyranoside
227		C_15_H_10_O_4_	Chrysophanic acid
228		C_10_H_20_O_6_	N-Butyl-β-D-fructoside
229		C_7_H_14_O_6_	Methyl-α-D-frutofuranoside
230		C_12_H_16_O_6_	Phenyl-β-D-glucopyranoside
231		C_13_H_18_O_6_	Benzyl-β-D-glucopyranoside
232		C_19_H_32_O_8_	Ascleposide E
233		C_19_H_12_O_8_	β-D-Frutofuranose
234		C_6_H_12_O_6_	Glucose
235		C_8_H_8_O_3_	Vanillin
236		C_6_H_6_O_3_	5-Hydroxymethyl-furaldehyde
237		C_9_H_10_O_4_	3,5-dimethoxy-4-hydroxy-benzaldehyde
238		C_21_H_38_O_4_	Monolinolein
239		C_4_H_6_O_4_	Succinic acid
240		C_17_H_32_O	Shikokiol A
241		C_17_H_32_O	Shikokiol B
242		C_17_H_32_O	Shikokiol C
243		C_7_H_6_O_2_	p-Hydroxybenzaldehyde
244		C_9_H_10_O_4_	3,5-Dimethoxy-4-hydroxyacetophenone
245		C_16_H_32_O_2_	Palmitic acid
246		C_10_H_14_	p-Cymene
247		C_19_H_34_O_2_	(Z, Z)-9,12-Octadecadienoic acid
248		C_21_H_38_O_4_	(Z, Z)-9,12-Octadecadienoic acid-2-hydroxy-1,3propamedinyl ester

### 5.1 Sesquiterpene lactones

The sesquiterpene lactones are the primary and characteristic metabolites of ALD, exhibiting a diverse and abundant range exceeding 130 species (Yuan et al., 2024; Zheng et al., 2022). Sesquiterpene lactones are a class of natural metabolites primarily found in plants of the Asteraceae family, characterized by a 15-carbon sesquiterpenoid backbone coupled with a lactone ring (Amen et al., 2025; Liu et al., 2021). They often serve as defensive metabolites in plants and are largely responsible for their characteristic bitter taste. These metabolites are renowned for their diverse and potent biological activities, including anti-inflammatory, anti-tumor, antimicrobial, and immunomodulatory effects (Amen et al., 2025). As a result, they represent not only key active metabolites in traditional herbal medicine but also important lead metabolites in modern drug development. However, their bioactivity is dual-edged: some members are strong allergens capable of inducing contact dermatitis and may exhibit cytotoxicity at higher concentrations (da Silva et al., 2021).

**FIGURE 2:**
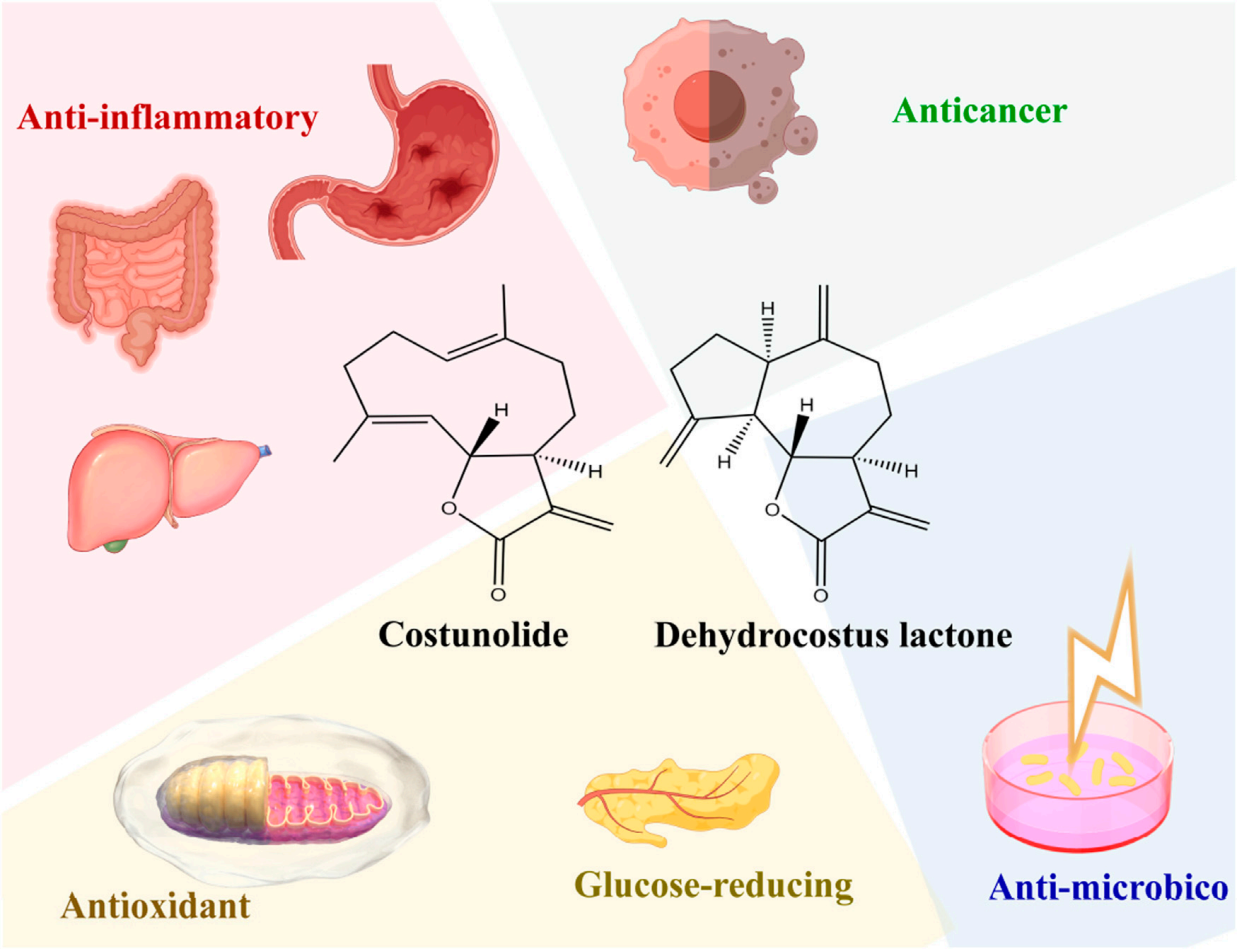
Bioactivities of dehydrocostus lactone and costunolide (by figdraw.com).

**FIGURE 3:**
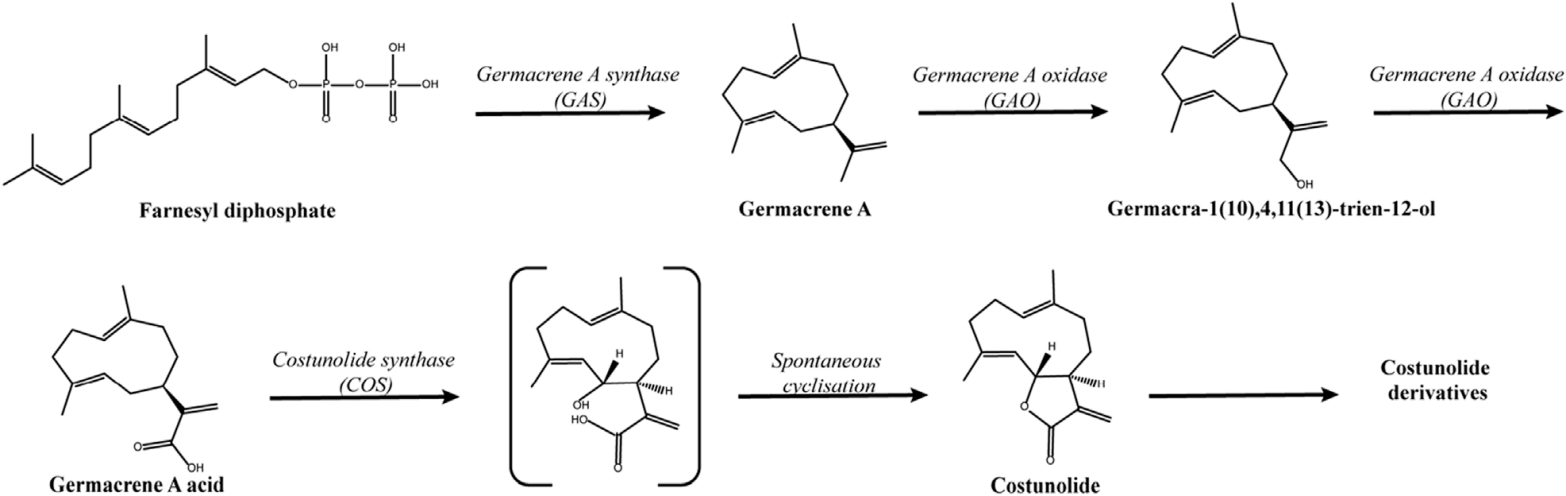
Biosynthetic pathway of costunolide.

Among the sesquiterpene lactones, costunolide and dehydrocostus lactone are the key substances for quality control of AR (Figure 2) (Dong et al., 2018). The total content of costunolide and dehydrocostus lactone in AR must not be less than 1.8%, as stipulated by the Chinese Pharmacopoeia (2020 Edition). They are also the most important active substances in AR and could be quantified synchronously using high-performance liquid chromatography coupled with mass spectrometry (Seo and Shin, 2015; Zhang et al., 2014). Currently, costunolide is already available in substantial quantities through genetic engineering techniques. The biosynthetic pathway for costunolide (Figure 3) has been successfully built in *Escherichia coli* by the co-expression of three genes (GAS, GAO, COS) involved in costunolide biosynthesis, along with eight genes responsible for converting acetyl-CoA into farnesyl diphosphate via the mevalonate pathway. And costunolide yield was up to 100 mg L^−1^ in *E. coli* (Yin et al., 2015). Meanwhile, the co-expression of GAS, GAO, and COS in yeast and *Nicotiana benthamiana* leaves has also facilitated costunolide production (Blazquez et al., 2011). Revealing the synthetic pathway of costunolide indicates that there are no obstacles at all in constructing transgenic plants of ALD that produce high yields of costunolide. However, despite the structural similarities between dehydrocostus lactone and costunolide, and their concurrent presence in plants, the biosynthetic pathway for dehydrocostus lactone in plants remains unidentified. Consequently, the production of dehydrocostus lactone still relies on the extraction from plant raw materials.

### 5.2 Monoterpenoids and triterpenoids

Monoterpenoids and triterpenoids are two important classes of natural terpenoid metabolites, each with distinct characteristics in structure, distribution, and function (Yang et al., 2025). Monoterpenoids, composed of two isoprene units (C10), are major metabolites of plant essential oils (Bhatti et al., 2014; Jiang and Wang, 2023). In contrast, triterpenoids consist of six isoprene units (C30), featuring higher molecular weight and greater structural complexity (Huang et al., 2024; Zeng et al., 2024). Monoterpenoids and triterpenoids are widely used in the flavor, food, and cosmetics industries. Their biological effects tend to be more profound and systemic, including notable anti-inflammatory, antitumor, immunomodulatory, and cholesterol-lowering activities (Yang et al., 2025). Representative metabolites such as α-amyrin and betulinic acid are core active metabolites in many traditional Chinese medicines. However, some triterpenoids may exhibit hepatotoxicity at high doses.

### 5.3 Phenylpropanoids

Phenylpropanoids are an important class of plant secondary metabolites characterized by a fundamental C6–C3 skeleton, which consists of a benzene ring (C6) attached to a propene group (C3), also known as the phenylpropane backbone (Vogt, 2010). These metabolites are primarily synthesized through the shikimate pathway and play crucial roles in plant defense, growth development, and signal transduction (Cao et al., 2025; Zhang et al., 2025). Additionally, they serve as significant sources for numerous pharmaceuticals, flavoring agents, and industrial raw materials.

### 5.4 Steroids

Steroids represent a significant class of bioactive metabolites in TCM. Phytosterols (β-sitosterol, lappalanasterol, pregnenolone, etc.) are the predominant type in ALD. Structurally, phytosterols resemble cholesterol in animals, sharing the same cyclopentanoperhydrophenanthrene core skeleton, but differ in their side chain configurations. These steroidal metabolites exhibit diverse structures and broad biological functions, serving not only as a fundamental chemical basis for explaining the efficacy and mechanisms of TCM, but also as key resources for modern drug (steroid hormones) development (Ferreira-Guerra et al., 2020).

### 5.5 Flavonoids

Flavonoids are ubiquitous in traditional Chinese medicine and are one of the key metabolites that enable many medicinal materials to exert their therapeutic effects. Flavonoids are distinguished by their broad spectrum of biological activities, including antioxidant effects, cardiovascular protection, and antiplatelet aggregation, which collectively contribute to reducing the risk of cardiovascular diseases and enhancing blood circulation (Jomova et al., 2025; Xu et al., 2025).

### 5.6 Others

In addition to the previously mentioned metabolites, ALD has been identified to contain polysaccharides, higher fatty acids, small aliphatic alcohols, aldehydes, acids, amino acids, and cholamine, among other metabolites (Peng et al., 2025; Zhuang et al., 2021). The presence of these metabolites in AR may provide a pharmacological basis for the utilization of this herbal medicine in the treatment of constipation, intestinal infections, and associated inflammatory diseases. Higher fatty acids, amino acids, and cholamine are essential for maintaining normal physiological functions of the body (Chen and Fang, 2025; Kelling et al., 2024; Wu et al., 2022). Meanwhile, although the specific mechanisms of action of aliphatic alcohols, aldehydes, and acids require further investigation, they may play significant roles in regulating metabolism and participating in immune responses.

## 6 Pharmacology

The pharmacological effects of AR are diverse and significant (Table 3).

**TABLE 3:** The main pharmacological properties of ALD.

Pharmacological activities	Main findings	References
Antioxidant	The preadministration of ALD extract exerted its protective effect of Th-induced adult male rats mainly through potentiating the antioxidant defense system by decreased lipid peroxidation and NO and increase the glutathione content.	Abdel-Rahman et al. (2020)
	The aqueous solvents of ALD were found superior in their ability to extract the antioxidants and aqueous ethanol was reported more efficient than aqueous methanol.	Ahmed et al. (2016)
	Alcoholic extract of AR (including alkaloids, terpenoid, phenols and others) have comparable antioxidant activity to ascorbic acid (81.96%).	Adel et al. (2025)
	The extract of ALD showed high levels of total phenolic content (188.2 ± 2.1 mg GAE/g DM) and total flavonoid content (129 ± 2.6 mg QE/g DM). In antioxidant tests, the extract exhibited strong activity, with the IC_50_ values of 137.15 μg/mL for ABTS and 175.5 μg/mL for DPPH.	Binobead et al. (2024)
	α,β-Unsaturated carbonyl metabolites (costunolide, dehydrocostus lactone, artemisitene, santamarine, isoalantolactone) were mainly featured as the antioxidant active metabolites of AR.	Wu et al. (2025)
	The 70% ethanol extract of ALD has a higher concentration of total phenolic content, total flavonoids, and antioxidant effect than the 70% methanol and water extracts. Rats pretreated with ALD extracts (70% methanol, 300 mg/kg BW) reduced the harmful effects induced by NaNO_2_ and improved the hematological parameters, liver, and kidney function biomarkers as well as lipid profile as compared to the NaNO_2_ group (75 mg/kg BW, single oral dose for 4 weeks).	Elshaer et al. (2022)
Anti-inflammatory	Dehydrocostuslactone exerts potent anti-hepatocellular carcinoma effects by inducing ER stress-mediated apoptosis via the MAPK pathway, resulting in a 50% reduction in tumor volume *in vivo* after 45 days of treatment.	Hsu et al. (2009)
	AR alleviates ulcerative colitis by targeting PKM2 to inhibit the NF-κB and NLRP3 pathways, thereby reducing inflammation and modulating immune responses.	Feng et al. (2024)
	Dehydrocostus lactone alleviates irinotecan (CPT-11)-induced intestinal mucositis by inhibiting the TLR4/MD2 complex and suppressing the NF-κB/NLRP3 signaling pathway, without compromising the antitumor efficacy of CPT-11.	Sun et al. (2024)
	The sesquiterpene lactone-rich fraction of ALD alleviates ulcerative colitis by regulating the Nrf2-Hmox-1, NF-κB, and MAPK pathways.	Chen et al. (2022a)
	ALD demonstrates potential in preventing and treating benign prostatic hyperplasia (BPH) by modulating apoptosis and inflammation, as evidenced by reduced prostate weight, improved apoptotic protein expression, and decreased inflammatory cytokines in a testosterone-induced BPH rat model.	Choi et al. (2021)
	The combined topical application of ALD and *Thuja orientalis* extracts demonstrates synergistic efficacy in alleviating atopic dermatitis symptoms by reducing pro-inflammatory activity and immune hyperresponsiveness, outperforming either extract alone.	Yang et al. (2017)
	α,β-Unsaturated carbonyl metabolites are as the key anti-inflammatory and antioxidant metabolites in ALD.	Wu et al. (2025)
	The sesquiterpenoids isolated from ALD, exhibit significant anti-inflammatory activity by inhibiting NO production in LPS-stimulated macrophages at 20 μM.	Lyu et al. (2023)
	In a DSS-induced murine ulcerative colitis UC model, daily gavage with dehydrocostus lactone at 20, 15, and 10 mg/kg/d from day 4–17 significantly reduced inflammation and enhanced barrier function by suppressing the IL-6/STAT3 pathway.	Zhou et al. (2020)
	Dehydrocostus lactone alleviates atherosclerosis by promoting cholesterol efflux and inhibiting inflammation via the TLR2/PPAR-γ/NF-κB signaling pathway in both *in vivo* and *in vitro* models.	Hong et al. (2025)
	Alantolactone exerts anti-inflammatory effects in LPS-stimulated macrophages by suppressing NF-κB activation and MAPK phosphorylation via downregulation of the MyD88-dependent signaling pathway.	Chun et al. (2012)
	Owing to the multi-target nature of IBD, the natural formulation KM1608 demonstrates potential therapeutic value by ameliorating colitis symptoms and distributing effectively in the intestinal tract.	Lee et al. (2020)
	Epoxymicheliolide alleviates ulcerative colitis by covalently targeting TAK1 and Keap1 to inhibit NF-κB-mediated inflammation and activate the Nrf2 antioxidant pathway.	He et al. (2022)
	Dehydrocostus lactone significantly ameliorated DSS-induced colitis in mice at doses of 5–15 mg/kg by covalently targeting both IKKα/β and Keap1, thereby suppressing NF-κB signaling and activating the Nrf2 pathway.	Yuan et al. (2022)
	ALD demonstrates potential as a therapeutic agent for osteoarthritis by exhibiting analgesic and anti-inflammatory effects in both *in vivo* and *in vitro* models.	Jo et al. (2021)
	The ethanol extract of ALD demonstrates anti-inflammatory effects by suppressing NF-κB and MAPK pathways and antioxidant activity through activation of the Nrf2/HO-1 pathway in LPS-stimulated RAW 264.7 cells.	Lim et al. (2020)
Anti-cancer effect	For the ethyl acetate extract of AR (including arbusculin B, α-cyclocostunolide, costunolide, and dehydrocostus lactone), cytotoxic IC_SOS_ of rat skeletal myoblast (L6 cells) from were from 1.6 to 19 μM, and selectivity indices from 0.5 to 6.5.	Julianti et al. (2011)
	The supercritical fluid extraction of oils from ALD obtained at 10 MPa exhibited the strongest antitumor efficacy with IC_50_ values of approximately 0.44, 0.46, and 0.74 μg/mL on HCT, MCF-7, and HepG-2 cells, respectively, whereas those at 20 MPa showed higher IC_50_ values (2.33, 6.59, 19.0 μg/mL), followed by 48 MPa (36.02, 59.5, 96.9 μg/mL).	Ahmed et al. (2022)
	Lyophilized ALD selectively inhibits the growth of breast and cervical cancer cells by inducing alternative apoptotic pathways.	Hasson et al. (2018)
	AR extract demonstrated significant antiproliferative and apoptotic effects, inducing caspase-3/7 and annexin V/PI activity, on MCF-7 breast cancer cells at concentrations of 20–200 μg/mL over 24–72 h, supporting its potential as a low-toxicity therapeutic candidate.	Kumar et al. (2024b)
	Stigmasterol, isoboldine, and β-sitosterol could target key prostate cancer-related hub genes (e.g., SRC, FGFR1, HSP90AA1); stigmasterol showed the strongest binding to HSP90AA1, and pathway analysis highlighted involvement of PI3K/AKT signaling.	Kosanam et al. (2024)
	A study on 72 mice with PVP-induced cancer demonstrated that AR extract exhibited anticancer effects by reducing cell proliferation and modulating liver enzyme levels (ALT: 29.01 ± 1.8, AST: 87.55 ± 2.9, ALP: 98.12 ± 8.8 U/L in controls), with dose-dependent tissue regeneration observed across treatment groups.	Al-Zayadi et al. (2023)
	AR extract at 500 mg/kg body weight demonstrated the highest anti-neoplastic efficacy in a DMBA-induced rat mammary tumour model, significantly reducing tumour progression, oxidative stress, pro-inflammatory cytokines (TNF-α and NF-κB), and expression of Ki-67, MMP-9, and VEGF markers.	Kumar et al. (2024a)
	AR extracts and isolated sesquiterpene lactones (particularly isoalantolactone, alantolactone, β-cyclocostunolide, and α-cyclocostunolide) exhibited significant cytotoxicity against A549 and C-6 cancer cells.	Kumar et al. (2014)
	The hexane and chloroform fractions of ALD exhibited potent anticancer activity against the PC-3 prostate cancer cell line, with IC_50_ values of 3.37 ± 0.14 μg/mL and 7.53 ± 0.18 μg/mL, respectively.	Bhushan et al. (2023b)
	ALD extracts demonstrated potent anticancer activity by inducing G1 phase arrest and intrinsic apoptosis via the mitochondrial pathway in breast, liver, and colon cancer cells, with IC_50_ values as low as 0.25–2.5 μg/mL for the most active extracts.	Shati et al. (2020)
	Three new eudesmane-type sesquiterpene lactone galactosides, costunosides A–C, were isolated from ALD and identified as the first natural β-galactopyranoside-containing eudesmane glycosides, among which metabolite 3 exhibited cytotoxic activity against several human cancer cell lines with IC_50_ values ranging from 3.4 to 9.3 µM.	Bhushan et al. (2023a)
	Dehydrocostus lactone demonstrates anti-angiogenic activity by inducing G0/G1 cell cycle arrest via inhibition of the Akt/GSK-3β/cyclin D1 and mTOR pathways, as evidenced in both *in vitro* and nude mice models.	Ahmad et al. (2012)
	Dehydrocostus lactone from ALD inhibits viability, migration, and proliferation of laryngeal carcinoma cells (Hep-2 and TU212) with low toxicity to normal HBE cells, and suppresses tumor growth *in vivo* by inducing mitochondrial apoptosis via inhibiting PI3K/Akt/Bad and activating ER stress pathways.	Zhang et al. (2020)
Organ protection	AR extract improved lung injury by reducing iNOS, caspase-3, and microRNA-let-7a, while boosting HO-1.	Attallah et al. (2023)
Anti-diabetic effect	Two proteinaceous amylase inhibitors, ScAI-R (IC50 = 23 μg/mL, Ki = 0.38 µM) and ScAI-L (IC50 = 28 μg/mL, Ki = 0.32 µM), which were from ALD, displayed the high affinities towards human salivary and pancreatic α-amylases (up to 90% inhibitory activity).	Ben Abdelmalek et al. (2025)
	The administration of extracts of ALD into Streptozotocin-treated rats separately resulted in a decline in the elevated levels of blood glucose, total cholesterol, triglycerides and improving serum HDL-Cholesterol and body weight.	Gomaa et al. (2021)
	All streptozotocin-treated diabetic rats received the treatments of ALD- extracts (200–400 mg/kg bwt., with isochlorogenic acid A (8393.64 μg/g) and chlorogenic acid (6,532.65 μg/g)) orally for 21 days consecutively, the administration significantly mitigated diabetic hyperglycemia.	Abouelwafa et al. (2024)
	The casein encapsulated extract of ALD demonstrated anti-diabetic potential through inhibition of α-amylase and α-glucosidase activities and enhanced glucose uptake in HepG2 cells.	Mushtaq et al. (2025)
Antiparasitic effect	The ethyl acetate extract of AR (including arbusculin B, α-cyclocostunolide, costunolide, and dehydrocostus lactone) potently inhibited the growth of *Trypanosoma brucei rhodesiense*, with ICsos between 0.8 and 22 μM.	Julianti et al. (2011)
	*Trichinella spiralis* experimental infection induced DNA damage and oxidative stress in rat skeletal muscles and treatments with AR extract modulates these changes.	Alghabban et al. (2024)
	*In vitro*, extract of AR showed a significant anthelmintic effect on live adult *Ascaridia.galli* worms in terms of inhibition of worm motility, with worm motility inhibition of 100% at 24 h post-exposure at the 100 mg/mL.	Mir et al. (2024)
Antimicrobial effect	The highest activity with the lowest MIC value was recorded as 3.12 μL/mL for the essential oil of ALD (against *S. epidermidis* and *C. albicans*), 3.12 mg/mL for the methanolic extract (against *S. aureus*), and 6.25 mg/mL for both hexane-chloroform and aqueous extracts (against *S. aureus*).	Ahmed and Coskun (2023)
	The smoke of ALD successfully inhibited *P. crustosum* growth of Fresh walnuts.	Qiao et al. (2023)
	Ethanol extracts of AR had inhibitory effects on common plant-pathogenic fungi, with EC50 (concentration for 50% of maximal effect) values ranging from 114.18 mg/L to 414.08 mg/L.	Cai et al. (2022)
	The highest minimum inhibitory concentration was seen in the ethanolic extract of ALD, with an MIC of 50 mg/mL for *S. aureus* followed by an MIC of 200 mg/mL for *K. pneumoniae*. It showed a MBC against *S. aureus* and *K. pneumoniae* (>50 mg/mL and >200 mg/mL, respectively).	(Mammate et al., 2023; Mishra et al., 2020)
	The acetic acid extract of ALD exhibited significant antimicrobial activity (*in vitro*) against *C. albicans* (MIC = 6.25 mg/mL, MFC = 12.5 mg/mL), followed by *B. cereus*, *S. enterica*, *S. aureus*, *E. coli*, and *P. aeruginosa*, respectively (MIC = 25 mg/mL, MBC/MFC = 25–50 mg/mL).	Idriss et al. (2023)
	The inhibitory activity of all ALD extracts at three different extraction pressure levels (10, 20, 48 MPa), was higher than gentamicin against all tested bacteria (*B. subtilis*, and *S. aureus*, *P. aeruginosa*, *E. coli*, *K. pneumonia*, *C. albicans*, *C. tropicalis*, *A. flavus*, *F. oxysporium*).	Ahmed et al. (2022)
Antiviral effect	The antiviral activity of ALD acetic acid extract had a significant positive influence against HSV-1 (EC50 = 82.6 g/mL; CC50 = 162.9 g/mL; selectivity index = 1.9). No effect was detected in terms of the inhibition of SARS-CoV2 entry.	Idriss et al. (2023)
	Some sesquiterpenoids isolated from ALD demonstrate anti-HBV activity by inhibiting HBsAg secretion.	Li et al. (2024a)
	The value of the *in vitro* IC50 of AR extract against low pathogenic human coronavirus (HCoV-229E) and human influenza virus (H1N1) influenza virus were 23.21 ± 1.1 and 47.6 ± 2.3 μg/mL, respectively.	Attallah et al. (2023)
	The ALD phytoconstituents have inhibitory potential against the receptor-binding domain of the spike glycoprotein and the main protease of the SARS-CoV-2 Delta (B.1.617.2) variant of the novel coronavirus via molecular docking, DFT, and ADME/Tox studies.	Houchi and Messasma (2022)
	The bioactive molecules from ALD can be as SARS-CoV-2 main protease inhibitors by computational approach investigation.	Hajji et al. (2022)

### 6.1 Antioxidant

The antioxidant profile of ALD multifaceted, stemming from its rich and diverse phytochemical composition. The high values for total phenolic (188.2 ± 2.1 mg GAE/g DM) and flavonoid (129 ± 2.6 mg QE/g DM) content are paramount (Binobead et al., 2024). Phenolics and flavonoids are renowned antioxidants due to their hydrogen-donating ability, which neutralizes free radicals by stabilizing them. The *in vitro* assays (DPPH and ABTS) confirm this radical-scavenging capability. The IC_50_ values (137.15 μg/mL for ABTS, 175.5 μg/mL for DPPH) represent the concentration required to scavenge 50% of the radicals (Binobead et al., 2024). Lower IC_50_ values indicate higher potency. The extract of AR have comparable antioxidant activity to ascorbic acid (Adel et al., 2025). α,β-Unsaturated carbonyl metabolites (costunolide, dehydrocostus lactone, artemisitene, santamarine, isoalantolactone) were mainly featured as the antioxidant active metabolites of AR (Wu et al., 2025). This molecular structure is a key pharmacophore for antioxidant activity. It can quench free radicals through direct single-electron transfer. Ultimately, the most significant evidence of its antioxidant power is its *in vivo* protective effect against NaNO_2_-induced toxicity. NaNO_2_ is a potent oxidant that causes methemoglobinemia and oxidative damage to organs. The extract’s ability to improve hematological parameters and protect liver and kidney function biomarkers demonstrates that its antioxidants are bioavailable and active within a living system, effectively mitigating systemic oxidative stress (Elshaer et al., 2022). This bridges the gap from laboratory findings to potential therapeutic applications, suggesting ALD could be developed into a natural remedy for conditions where oxidative stress is a key pathological factor.

### 6.2 Anti-inflammatory

The extract of ALD and its isolated bioactive metabolites, particularly sesquiterpene lactones such as dehydrocostus lactone, alantolactone, and epoxymicheliolide, demonstrate significant anti-inflammatory activity across multiple experimental models. In LPS-stimulated RAW 264.7 macrophages and peritoneal macrophages, ALD and these metabolites consistently suppress the production of key proinflammatory mediators, including NO, PGE2, iNOS, COX-2, and the cytokines IL-6, IL-1β, and TNF-α (He et al., 2022; Lim et al., 2020). This broad inhibition of inflammatory outputs is not limited to macrophage models but extends to *in vivo* conditions such as osteoarthritis (Jo et al., 2021), where ALD reduces pain and serum IL-1β, and to inflammatory bowel disease (IBD) models (Lee et al., 2020), including dextran sulfate sodium (DSS)-induced colitis (Yuan et al., 2022), where it ameliorates symptoms, protects the colonic barrier, and lowers inflammatory cytokines and enzymes like myeloperoxidase (MPO).

**FIGURE 4:**
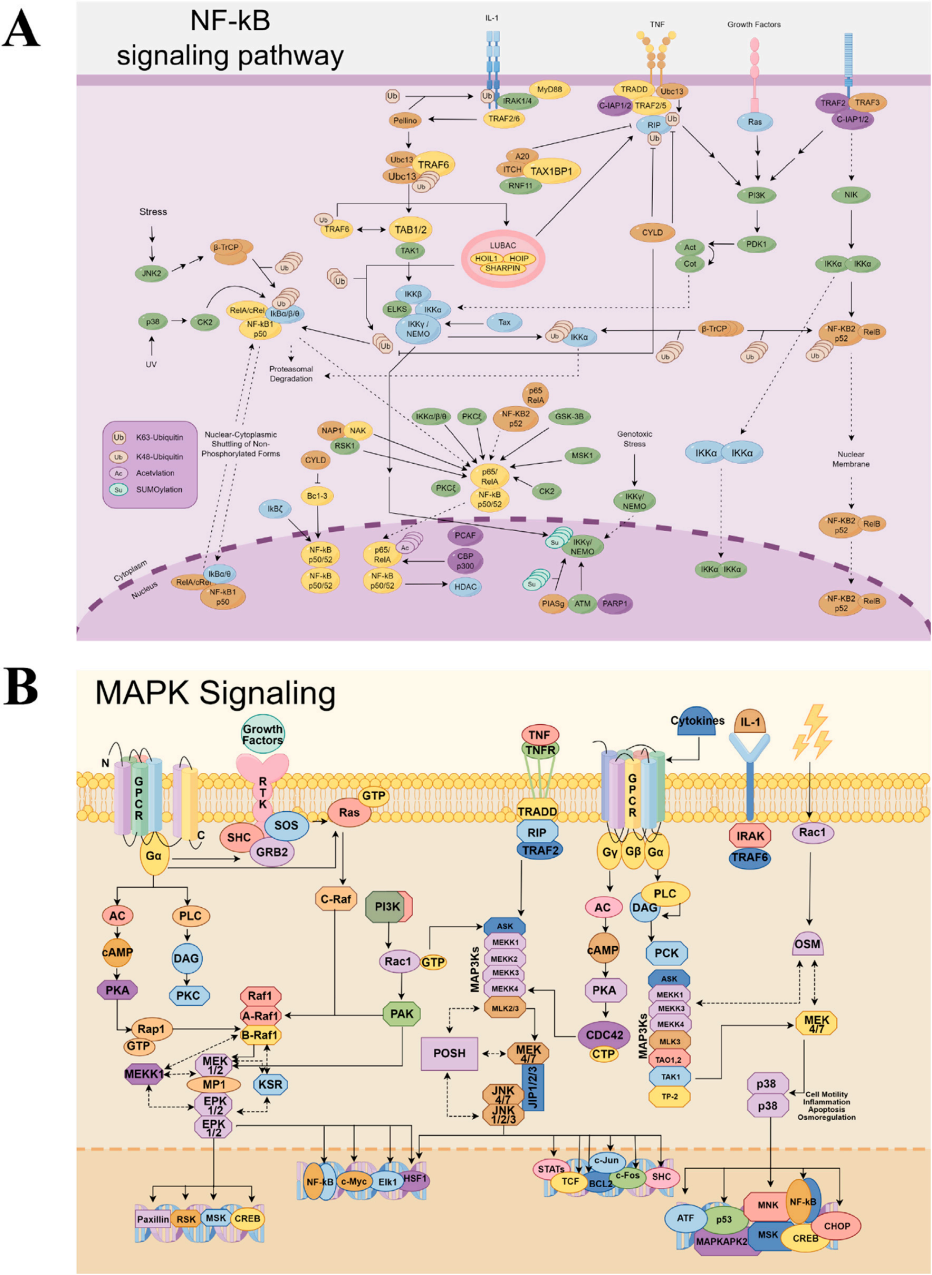
NF-κB **(A)** and MAPK **(B)** signaling pathways (by figdraw.com).

The fundamental mechanism underlying this robust anti-inflammatory effect involves the dual suppression of the NF-κB and MAPK signaling pathways (Figure 4) (Lim et al., 2020). ALD and its metabolites inhibit the LPS-induced phosphorylation and degradation of IκBα, thereby preventing the nuclear translocation of the NF-κB subunits p65 and p50 (Chun et al., 2012; Lim et al., 2020). Concurrently, these metabolites inhibit the phosphorylation of MAPKs, including JNK, ERK, and p38 (Chun et al., 2012; Hsu et al., 2009; Lim et al., 2020). Further upstream, alantolactone was shown to suppress the expression of adaptor proteins MyD88 and TIRAP, which are critical for initiating these cascades (Chun et al., 2012). Importantly, this anti-inflammatory activity is complemented by a potent antioxidative effect mediated through the activation of the Nrf2/HO-1 pathway. Metabolites like dehydrocostus lactone and epoxymicheliolide enhance the nuclear accumulation of Nrf2, leading to the upregulation of antioxidant genes and a reduction in intracellular ROS (Yuan et al., 2022). Mechanistic studies reveal that many of these actions depend on the presence of an α,β-unsaturated carbonyl group, which allows the metabolites to form covalent bonds with key cysteine residues on target proteins like IKKα/β, Keap1, or TAK1 (He et al., 2022; Wu et al., 2025; Yuan et al., 2022).

The therapeutic implications of these mechanisms are vast, as evidenced by efficacy in diverse inflammatory disease models. Beyond colitis and osteoarthritis, ALD and dehydrocostus lactone alleviate atherosclerosis by promoting cholesterol efflux in macrophage-derived foam cells and modulating the TLR2/PPAR-γ/NF-κB pathway (Hong et al., 2025). They also show protective effects against irinotecan-induced intestinal mucositis by inhibiting the TLR4/NF-κB/NLRP3 axis and against benign prostatic hyperplasia by restoring apoptosis balance (Choi et al., 2021; Sun et al., 2024). Furthermore, a sesquiterpene lactone-rich fraction of ALD was significantly more effective than an aqueous extract in treating ulcerative colitis (Chen Y. et al., 2022), underscoring that these specific metabolites are the primary active anti-inflammatory agents. This multifaceted, multitargeted action, targeting both inflammation and oxidative stress, positions ALD and its metabolites as promising candidates for treating complex chronic inflammatory disorders.

### 6.3 Anti-cancer effect

The supercritical fluid extraction of oils from ALD demonstrates remarkable and concentration-dependent cytotoxic efficacy against various cancer cell lines. Specifically, the extract obtained at 10 MPa exhibited potent antitumor activity with IC_50_ values of approximately 0.44, 0.46, and 0.74 μg/mL against HCT-116, MCF-7, and HepG-2 cells, respectively, whereas extracts obtained at higher pressures (20 MPa and 48 MPa) showed significantly reduced potency, highlighting the critical influence of extraction parameters on bioactive metabolite efficacy (Ahmed et al., 2022). Furthermore, specific sesquiterpene lactones like costunolide, dehydrocostus lactone, alantolactone, and isoalantolactone, isolated from the roots, have demonstrated significant activity against diverse cancer types including lung (A549), glioma (C-6), and prostate (PC-3) cancer cells, with hexane and chloroform fractions of ALD showing particularly low IC_50_ values, such as 3.37 ± 0.14 and 7.53 ± 0.18 μg/mL against PC-3 cells, respectively (Bhushan et al., 2023b; Kumar et al., 2014).

The anticancer mechanisms of these extracts and metabolites are primarily mediated through the induction of apoptosis via both intrinsic and extrinsic pathways. Lyophilized ALD significantly suppressed the growth and proliferation of T-47D and HeLa cells by inducing apoptosis, as evidenced by suppressed LDH release, reduced NO production, and activation of death receptors in a dose-dependent manner (Hasson et al., 2018). Similarly, ALD extract promoted apoptosis in MCF-7 cells by activating caspase-3/7 and annexin V/PI pathways (Kumar et al., 2024b). In-depth mechanistic studies on dehydrocostus lactone have revealed its ability to inhibit angiogenesis by inducing G0/G1 cell cycle arrest in human umbilical vein endothelial cells (HUVECs) through the abrogation of the Akt/GSK-3β/cyclin D1 and mTOR signaling pathways (Ahmad et al., 2012). Moreover, in laryngeal carcinoma cells, dehydrocostus lactone induced mitochondrial apoptosis by inhibiting the PI3K/Akt/Bad pathway and stimulating endoplasmic reticulum stress-mediated apoptosis, accompanied by the upregulation of p53 and P21 (Zhang et al., 2020).

**FIGURE 5:**
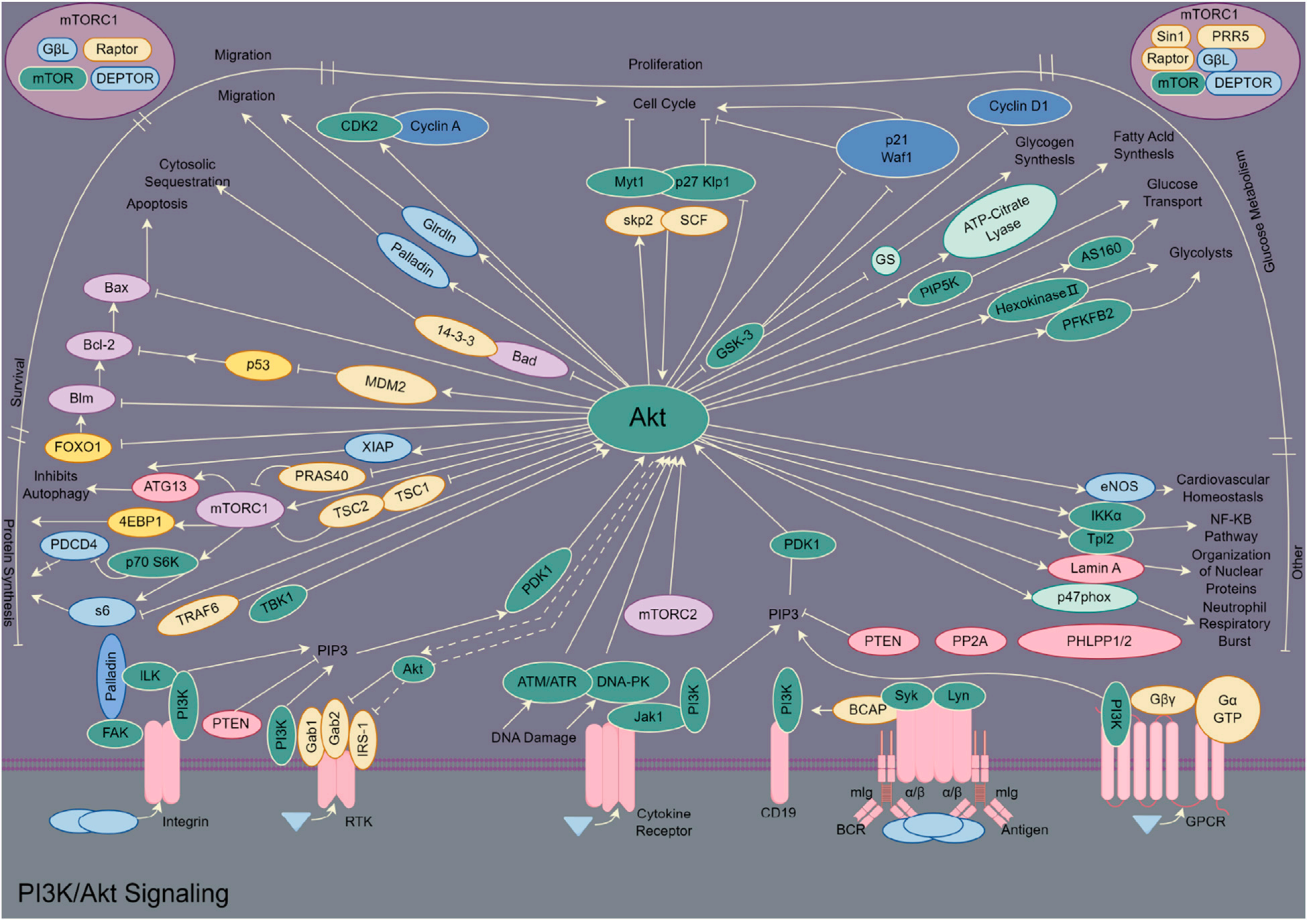
PI3K/Akt signaling pathway (by figdraw.com).

*In vivo* studies substantiate the anticancer potential and reduced systemic toxicity of these natural metabolites. In a 12-dimethylbenz (a) anthracene (DMBA)-induced mammary tumour model in rats, treatment with ALD root extract at 500 mg/kg body weight resulted in significant chemopreventive effects, demonstrated by inhibition of tumour parameters, minimal alterations in liver and kidney enzymes, reduction in oxidative stress, decreased pro-inflammatory cytokines (TNF-α and NF-κB), and downregulation of proliferation (Ki-67), metastasis (MMP-9), and angiogenesis (VEGF) markers (Kumar et al., 2024a). Another study in mice induced with cancer using Polyvinyl pyrrolidone K-30 (PVP) showed that ethanolic extract of ALD reduced cancer cell proliferation and mitigated histopathological damage in the liver and kidneys in a dose-dependent manner, contrasting with the severe damage observed in chemotherapy-treated groups (Al-Zayadi et al., 2023). Furthermore, molecular docking and virtual screening studies identified bioactive metabolites in ALD, such as stigmasterol, isoboldine, and beta-sitosterol, with favourable ADME (Absorption, Distribution, Metabolism, and Excretion) and toxicity profiles, showing strong binding affinities to hub genes like SRC, FGFR1, and HSP90AA1 involved in prostate cancer pathways, particularly PI3K/AKT signaling (Figure 5) (Kosanam et al., 2024). This multi-targeted approach, combined with a favourable safety profile, positions ALD and its bioactive metabolites as promising candidates for further development as anticancer therapeutics.

### 6.4 Organ protection

AR extract demonstrates significant organ-protective effects, particularly against acute lung injury, through a multi-target and multi-pathway mechanism. The study revealed that AR extract ameliorates cyclophosphamide-induced histological damage in the lung by concurrently modulating inflammatory, oxidative, and apoptotic pathways (Attallah et al., 2023). Specifically, its protective action is mediated through the reduction of iNOS and the caspase-3, which attenuates excessive inflammation and inhibits programmed cell death, respectively. Furthermore, AR extract alleviates oxidative stress by significantly decreasing the level of MDA, a marker of lipid peroxidation, while upregulating the gene expression of the HO-1. The accompanying downregulation of microRNA-let-7a suggests a potential involvement in epigenetic regulation, though its precise role requires further elucidation. This evidence collectively indicates that AR extract’s organoprotective efficacy is achieved via a synergistic combination of anti-inflammatory, antioxidant, and anti-apoptotic activities.

### 6.5 Anti-diabetic effect

ALD exhibits multi-faceted anti-diabetic properties, demonstrated through both *in vitro* and *in vivo* studies. Two novel non-competitive proteinaceous inhibitors, ScAI-R (IC_50_ = 23 μg/mL, Kᵢ = 0.38 µM) and ScAI-L (IC_50_ = 28 μg/mL, Kᵢ = 0.32 µM), purified from the roots and leaves, showed high affinity towards human salivary and pancreatic α-amylases, achieving up to 90% inhibitory activity (Ben Abdelmalek et al., 2025). Further supporting these findings, the casein-encapsulated extract of bioactive metabolites from ALD significantly inhibited α-amylase and α-glucosidase activities and enhanced glucose uptake in HepG2 cells (Mushtaq et al., 2025). In diabetic rat models induced by Streptozotocin, oral administration of ALD extracts—containing high concentrations of phenolic metabolites such as dehydrocostus lactone, azulene, eicosapentaenoic acid, linoelaidic acid, isochlorogenic acid A, and chlorogenic acid resulted markedly reduced blood glucose, total cholesterol, and triglyceride levels, while improving HDL-cholesterol and body weight (Abouelwafa et al., 2024; Gomaa et al., 2021). These results collectively highlight the potential of ALD a source of effective anti-diabetic agents through enzyme inhibition and metabolic regulation.

### 6.6 Antiparasitic effect

Based on multiple studies, the extract of ALD and its specific bioactive metabolites, such as costunolide and dehydrocostuslactone, demonstrate broad-spectrum antiparasitic properties by exhibiting both direct lethal effects and indirect host-protective mechanisms. The ethyl acetate extract of AR (including arbusculin B, α-cyclocostunolide, costunolide, and dehydrocostuslactone) potently inhibited the growth of Trypanosoma brucei rhodesiense, with ICsos between 0.8 and 22 μM (Julianti et al., 2011). *In vitro*, extract of AR showed a significant anthelmintic effects on live adult *Ascaridia.galli* worms in terms of inhibition of worm motility, with worm motility inhibition of 100% at 24 h post-exposure at the 100 mg/mL (Mir et al., 2024). Beyond direct parasite killing, research reveals a complementary protective role; in a Trichinella spiralis-infected rat model, the extract significantly modulated infection-induced DNA damage and oxidative stress in host tissues (Alghabban et al., 2024). This collective evidence positions ALD as a highly promising source for novel antiparasitic agents, offering a dual mechanism of action that combines direct potency with alleviation of the pathological damage caused by parasitic infections.

### 6.7 Antimicrobial effect

The essential oil, hexane-chloroform, methanolic, and aqueous extracts of AR have been proven to have certain antimicrobial effects on *Acinetobacter baumannii*, *Aspergillus flavus*, *Alternaria alternata*, *Bacillus cereus*, *Blumeria graminis*, *Botrytis cinerea*, *Candida tropicalis*, *Colletotrichum gloeosporioides*, *Candida albicans*, *Didymella glomerata*, *Escherichia coli*, *Enterobacter cloacae*, *Enterococcus faecalis*, *Fusarium oxysporum*, *Fusarium graminearum*, *Fusarium lateritium*, *Klebsiella pneumonia*, *Pseudomonas aeruginosa*, *Pythium aphanidermatum*, *Phytophthora infestans*, *Sclerotinia sclerotiorum*, *Salmonella enterica*, *Staphylococcus aureus*, and *Staphylococcus epidermidis* (Ahmed and Coskun, 2023; Ahmed et al., 2022; Cai et al., 2022; Idriss et al., 2023). Meanwhile, the smoke of ALD successfully inhibited *Penicillium crustosum* growth of fresh walnuts (Qiao et al., 2023). The antimicrobial mechanism of the extract of AR is multi-targeted, mainly including: damaging the structure of the cell membrane, inhibiting the formation of biofilms, inhibiting quorum sensing and inducing the production of ROS. The composites of ALD demonstrated significantly stronger antimicrobial activity compared to its individual metabolites, causing clear structural damage to resistant strains, such as chitosan-AR nanoconjugates (Alshubaily, 2019), iron oxide nanoparticles of AR (Al-Shaeri and Al-brahim, 2023), and AR-MgO nanoparticles (Mishra et al., 2020). AR extract is undoubtedly a natural antibacterial agent with great research and development value. Its broad-spectrum antibacterial activity, especially its effective effect on drug-resistant bacteria and biofilms, makes it a promising candidate for addressing the global antibiotic resistance crisis.

### 6.8 Antiviral effect

Studies have demonstrated the broad-spectrum antiviral potential of extracts and metabolites from ALD and related botanicals. Some lappanolides from ALD exhibits excellent anti-HBV activity (Li H.-B. et al., 2024). The value of the *in vitro* IC50 of AR extract against low pathogenic human coronavirus (HCoV-229E) and human influenza virus (H1N1) influenza virus were 23.21 ± 1.1 and 47.6 ± 2.3 μg/mL, respectively. (Attallah et al., 2023). The antiviral activity of ALD acetic acid extract had a significant positive influence against HSV-1 (EC50 = 82.6 g/mL; CC50 = 162.9 g/mL; selectivity index = 1.9) (Idriss et al., 2023).

The bioactive molecules from ALD can be as SARS-CoV-2 main protease inhibitors by computational approach investigation (Hajji et al., 2022). The ALD phytoconstituents have inhibitory potential against the receptor-binding domain of the spike glycoprotein and the main protease of the SARS-CoV-2 Delta (B.1.617.2) variant of the novel coronavirus using molecular docking, DFT, and ADME/Tox studies (Houchi and Messasma, 2022). However, in the actual experiment, no effect was still detected in terms of the inhibition of SARS-CoV2 entry (Idriss et al., 2023).

Currently, the vast majority of research on the antiviral effects of ALD (especially against SARS-CoV-2) remains at the stage of computer simulations (e.g., molecular docking) and *in vitro* studies. While these findings provide a solid theoretical foundation and valuable guidance for further investigation, there is still a long way to go before clinical application can be realized. Validation through animal studies and human clinical trials is still needed. It is also important to note that ALD a complex multi-metabolite system, and its antiviral activity likely results from the synergistic effects of various metabolites rather than a single metabolite. From a modern scientific perspective, its antiviral properties may be associated with holistic regulatory functions, such as anti-inflammatory and immunomodulatory effects.

## 7 Conclusion

In the-present review, we have-presented the biological characteristics, cultivation techniques, chemical composition, pharmacological activities, and processing techniques pertaining to ALD. ALD, as core metabolites in traditional Chinese herbal formulations for treating hepatic and intestinal inflammation, have drawn global attention for their therapeutic potential in biopharmaceutical applications. The future development of ALD-derived products must be propelled by technology-driven innovation and supported by institutional synergy. Through standardized systems to elevate industrial upgrading, regulatory science to dismantle market barriers, synthetic biology to redefine resource supply, and clinical research to unlock health potential, ALD-related industries are poised to emerge as global benchmarks in biopharmaceuticals and sustainable development. This transformation will not only alleviate global pressures on natural drug supply chains but also disseminate the wisdom of traditional Chinese medicine worldwide, advancing the modernization of traditional Chinese medicine into the global health governance framework.

## 8 Further perspectives

### 8.1 Limitations

Although ALD demonstrates significant medicinal potential, particularly its notable anticancer activity, there remain major limitations in current research that severely hinder its transition from a traditional remedy to a modern, internationally recognized drug.

First, the standardization of botanical drug sources and quality is the primary obstacle. The origin of ALD is complex, and variations in harvesting seasons and processing methods lead to significant differences in the types and concentrations of its internal chemical metabolites. Existing research has predominantly focused on exploring the activity of specific extracts or metabolites but lacks a comprehensive and systematic quality evaluation system for the botanical drug itself. Most studies rely only on individual active metabolites (such as costunolide and dehydrocostus lactone) as quality control indicators, which fails to comprehensively reflect the holistic nature of the botanical drug and its “multi-metabolite, multi-target” mechanism of action. This lack of standardization makes it difficult to reproduce, compare, and integrate research results from different laboratories, resulting in fragmented data that cannot provide a consistent and reliable material basis for clinical medication.

Secondly, research on the active metabolites and their mechanisms of action remains insufficient. Although numerous bioactive metabolites (costunolide, dehydrocostus lactone, alantolactone, etc.), have been isolated and identified, and their functions in inducing apoptosis and inhibiting angiogenesis have been confirmed, most studies remain at the stage of phenomenological observation and preliminary mechanistic exploration. The majority of mechanistic research relies on *in vitro* cell models, where the drug concentrations used are often significantly higher than the achievable levels *in vivo*, raising doubts about the extrapolation of the findings. There is a notable lack of research on how the multiple metabolites in ALD interact synergistically or antagonistically to produce therapeutic effects, which is an aspect central to the philosophy of traditional Chinese medicine. Furthermore, current studies focus predominantly on its anticancer properties, while modern scientific explanations of its traditional efficacy are severely lacking. Little effort has been made to integrate newly discovered functions (e.g., anti-inflammatory effects and regulation of gastrointestinal motility) with traditional knowledge.

Thirdly, the pharmacokinetic and safety evaluation systems require significant improvement. The processes of oral absorption, distribution, metabolism, and excretion of the main active metabolites in ALD, such as lactones, remain largely unclear. Key questions persist: What is their bioavailability? In what form do they exert effects *in vivo*, as parent metabolites or metabolites? What kind of pharmacokinetic interactions exist between these metabolites and commonly used chemotherapy drugs? All these questions remain unanswered. Although some studies suggest that its extracts exhibit low toxicity to normal cells, there is a lack of systematic toxicological studies that meet modern drug approval standards, including investigations into long-term toxicity, reproductive toxicity, and genotoxicity. The potential risk of “nephrotoxicity” has also been frequently mentioned, but solid experimental data to either confirm or refute this claim are still lacking. This uncertainty represents a major concern for its clinical adoption.

Finally, there is a significant lack of clinical research. Almost all encouraging data currently available come from preclinical studies (*in vitro* cell and animal experiments). There is a shortage of rigorously designed and standardized clinical trials to verify the actual efficacy and safety of these treatments in humans. In traditional Chinese clinical practice, ALD is most commonly used in formulations. However, modern research has rarely attempted to simulate such complex environments to explore its role and effects within these formulations. Moreover, the considerable gap between discovering highly active extracts or metabolites in the laboratory and developing them into quality-controlled, standardized preparations suitable for patient use remains largely unaddressed.

### 8.2 Future research needs

To promote the in-depth development of ALD research and facilitate its clinical translation, future work should focus on the following aspects to meet the needs of a comprehensive research pipeline from basic to applied studies.

Firstly, future research needs to focus on establishing a quality control methodology based on a “holistic perspective”. Modern chromatographic, spectroscopic, and coupling techniques (such as HPLC-MS and GC-MS) should be utilized, in combination with chemometric methods, to conduct systematic analysis of ALD from different sources. This will help establish a “fingerprint profiling” standard that covers multiple major active metabolites and characteristic metabolites. At the same time, active research on spectrum-effect relationships should be carried out to correlate chemical fingerprints with pharmacological efficacy data. This will help identify which groups of metabolites are the key material basis for specific pharmacological effects (such as anti-cancer, anti-inflammatory, and anti-ulcer activities).

Secondly, it is essential to employ multi-omics technologies (genomics, transcriptomics, proteomics, metabolomics) and systemic biological approaches to comprehensively and unbiasedly uncover the target sites and network pathways of ALD and its active metabolites. This represents a core requirement. Comprehensive clinical studies in line with international standards must be initiated. It is imperative to systematically complete pharmacokinetic studies on the main active metabolites and optimized extracts of ALD to clarify their ADME properties. Additionally, toxicological evaluations should be conducted to thoroughly assess the safety of long-term usage and fully elucidate potential risks such as nephrotoxicity.

Thirdly, innovative formulation technologies and translational research should be a top priority for future studies. Given that many lactone metabolites exhibit poor water solubility and low bioavailability, there is a need to develop novel drug delivery systems such as nanoparticles, liposomes, and phospholipid complexes, to enhance their targeting capability and therapeutic efficacy. Furthermore, these advanced systems can help mitigate systemic toxicity associated with non-selective drug exposure.

### 8.3 Priorities

Faced with numerous research demands, it is crucial to concentrate resources on addressing key issues. In the short term, priority should be given to foundational work on quality standardization: immediately launching a large-scale chemical composition survey of mainstream commercial ALD, integrating genomics for origin identification, establishing rapid and accurate identification methods based on multi-metabolite quantitative analysis and DNA barcoding technology, and formulating unified and feasible standards for raw medicinal materials. The most promising core active metabolites should be prioritized, with resources focused on completing systematic preclinical pharmacokinetic and pharmacodynamic studies, as well as preliminary acute toxicity and repeated-dose toxicity tests, to quickly obtain an initial assessment of their safety risks. Meanwhile, advanced technologies such as CRISPR screening, molecular docking, and metabolite probes should be employed to precisely identify their direct target sites.

Research on the mechanisms of compatibility in TCM formulations is also one of the priorities. Conducting studies on the synergistic principles and toxicity-reducing effects of pairing ALD with other botanical drugs is essential to provide a scientific basis for the modern application of TCM formulas. For compounds that have been identified as highly active but with low bioavailability, it is necessary to initiate the development of novel drug delivery systems, such as preparing their nanocrystals or phospholipid complexes, and to validate their efficacy enhancement and toxicity reduction in animal models. Additionally, launching small-scale exploratory clinical studies on TCM will help accumulate preliminary data and experience for larger trials.

The establishment of a complete industrial chain can maximize the internationalization of the ALD industry. Facilitating the formation of an integrated industrial chain, which is from large-scale cultivation of ALD bases to standardized extraction and production, and further to the development of final formulations, will help translate research achievements into real industrial applications and build a modern traditional Chinese medicine brand. Meanwhile, establishing an ALD research database to integrate chemical, pharmacological, toxicological, and clinical data will promote global collaboration and knowledge sharing among researchers.

## Abbreviations

ALD, Aucklandia lappa Decne.; AR, Aucklandiae Radix; ARR, Aristolochiae Radix; DSS, dextran sulfate sodium; E-nose, electronic nose; GC, gas chromatography; IBD, inflammatory bowel disease; ICPMS, inductively coupled plasma mass spectrometry; IR, Inulae Radix; LC, liquid chromatography; MBC, minimal bactericidal concentration; MIC, minimum inhibitory concentration; MS, mass spectrometry; NMR, nuclear magnetic resonance; TCM, traditional Chinese medicine; TLC, thin-layer chromatography; VR, Vladimiriae Radix.
